# Differential modulation of visual responses by distractor or target expectations

**DOI:** 10.3758/s13414-022-02617-w

**Published:** 2022-12-02

**Authors:** M. P. Noonan, A. H. Von Lautz, Y. Bauer, C. Summerfield, M. S. Stokes

**Affiliations:** 1 Department of Experimental Psychology, University of Oxford, Oxford, UK; 2 Division of Neurobiology, Faculty of Biology, LMU Munich, 82152 Munich, Germany; 3 Graduate School of Systemic Neurosciences (GSN), LMU Munich, 82152 Munich, Germany

**Keywords:** Distractor suppression, Expectation, P1, N2pc, Frontocentral theta, Alpha, Decoding

## Abstract

Discriminating relevant from irrelevant information in a busy visual scene is supported by statistical regularities in the environment. However, it is unclear to what extent immediate stimulus repetitions and higher order expectations (whether a repetition is statistically probable or not) are supported by the same neural mechanisms. Moreover, it is also unclear whether target and distractor-related processing are mediated by the same or different underlying neural mechanisms. Using a speeded target discrimination task, the present study implicitly cued subjects to the location of the target or the distractor via manipulations in the underlying stimulus predictability. In separate studies, we collected EEG and MEG alongside behavioural data. Results showed that reaction times were reduced with increased expectations for both types of stimuli and that these effects were driven by expected repetitions in both cases. Despite the similar behavioural pattern across target and distractors, neurophysiological measures distinguished the two stimuli. Specifically, the amplitude of the P1 was modulated by stimulus relevance, being reduced for repeated distractors and increased for repeated targets. The P1 was not, however, modulated by higher order stimulus expectations. These expectations were instead reflected in modulations in ERP amplitude and theta power in frontocentral electrodes. Finally, we observed that a single repetition of a distractor was sufficient to reduce decodability of stimulus spatial location and was also accompanied by diminished representation of stimulus features. Our results highlight the unique mechanisms involved in distractor expectation and suppression and underline the importance of studying these processes distinctly from target-related attentional control.

**Supplementary Information:**

The online version contains supplementary material available at 10.3758/s13414-022-02617-w.

## Introduction

Information processing depends on explicit and implicit factors. Explicit factors that affect stimulus processing include preparative cueing, top-down attentional control settings, and context control signals (Fogelson & Fernandez-Del-Olmo, 2013; Folk et al., 1992; Posner, 1980; Ruff & Driver, 2006). Neurobiologically, many of these factors result in facilitative processes (Desimone & Duncan, 1995), which in the case of goal-relevant stimuli (targets) improve behavioural performance. By contrast, implicit factors depend on how we process environmental statistics of our historical experiences (Awh et al., 2012). These processes can affect goal-relevant and goal-irrelevant stimuli (distractors), with prominent neurobiological theories, such as repetition suppression or expectation suppression, arguing reduced neural activity is associated with increased experiences of stimuli (Henson, 2003; Summerfield et al., 2008). Target processing is affected by statistics of target-related information, which can often lead to higher discriminability (Summerfield & de Lange, 2014), but it can also be modulated by the presence and features of the distractor, such as adjusting the representation of the target to maximize discriminability from the distractor (Geng et al., 2017; Yu & Geng, 2019). While top-down models of explicit factors have often been investigated, we know less about how trial-wise target processes are affected by implicit processes, particularly distractor-related experience.

To address this, the current study had two aims. First, to determine the degree to which stimulus repetition, of either targets or distractors, and higher order expectations, are supported by the same neural mechanisms. Experimentally, stimulus repetition is often difficult to disentangle from increasing ‘higher order’ expectations. Higher order expectations go beyond the physical properties of the stimulus itself and reflect the probability that a repetition will occur which is itself dependent on the stimuli which occurred further back in the stimulus history (see Grotheer & Kovacs, 2016, for extensive discussion). For example, behavioural performance is dependent on the spatial or feature-based predictability of the distractor (Feldmann-Wustefeld & Schubo, 2016; Leber et al., 2016; Reder et al., 2003, Sauter et al., 2018, Wang & Theeuwes, 2018a, 2018b), with effects showing a spatial gradient from the predicted site (Sauter et al., 2018) and can even be implicitly tied to the location of the target stimuli (Leber et al., 2016). While few studies to date have examined the neural mechanisms underlying distractor expectations, early evidence suggests reduced distractor-specific processing in ERP components (van Moorselaar et al., 2020; van Moorselaar & Slagter, 2019).

The second aim of the present study is to examine whether the neural mechanisms supporting target and distractor-related processes after stimulus repetition or higher order expectation are distinguishable. This aim builds on previous work that has shown target discriminability is improved, and the cost of a competing distractor reduced, when distractors are repeated in the same location (Noonan et al., 2016, Wang & Theeuwes, 2018a, 2018b). Others have shown similar effects in repeated experience of a particular distractor feature, such as colour (Cunningham & Egeth, 2016; Lamy et al., 2008). Repeated distractors also appear to be subjected to the same neural suppression mechanisms to reduce stimulus-evoked neural activity as targets (Henson, 2016) with evidence of a diminished P1 contralateral to the distractor and a reduction in the lateralized N2pc component (Noonan et al., 2016) in blocks of trials in which a distractor repeats to the same location. Suppression of the P1 is also associated with inhibition of repeated distractor features such as colour (Moher et al., 2014). While relatively complementary processes appear to support target and distractor repetition effects, the mechanisms supporting higher order target and distractor expectations are unclear.

To investigate the behavioural and neural mechanisms associated with target and distractor repetition and expectation, we modified our previous four-location stimulus discrimination paradigm by implicitly varying the spatial predictability of targets and distractors across time. The two stimuli could either be fully predictable to a spatial location (100%), be highly expected (75%), or appear randomly (25%) in each location, examining both behavioural and neural measures derived from EEG and MEG. We predicted improved behavioural performance following a repeat target or distractor (as shown previously; Noonan et al., 2016) as a function of increased expectations of either target or distractor.

With our results confirming these predictions and suggesting a remarkable similarity in the interactive influence of these two factors on behaviour, we examined whether the neural activity, as measured by EEG, would identify similar neural mechanisms or whether the more temporally resolved analyses would differentiate stimulus processing. We examined broadband ERP activity and showed that spatial repetitions predominantly accounted for variance in early visual event-related components such as the P1 and N2pc, with targets and distractors having opposing influence on the direction of these components

Critically, we also examined the modulation of time frequency decomposed stimulus-evoked alpha and theta power by expected and unexpected stimulus repeats. With oscillatory power in the alpha range inversely related to other measures of neural activity (Bonnefond & Jensen, 2012; Haegens et al., 2011; Laufs, Kleinschmidt et al., 2003; Spaak et al., 2012) as well as visual excitability (Hanslmayr et al., 2007; Myers et al., 2014; Romei et al., 2008, van Dijk et al., 2008,), increased alpha power has repeatedly been associated with stimulus suppression (Klimesch, 2012; Worden et al., 2000) and attentional gating mechanisms (Bonnefond & Jensen, 2015; Jensen & Mazaheri, 2010). Stimulus evoked alpha power is also modulated by expectation, with reduced alpha when expectations are violated (Rungratsameetaweemana et al., 2018). By contrast, frontocentral theta power is increased when expectations are violated (Rungratsameetaweemana et al., 2018). Theta power also differentiates expected target repetition and unexpected repetitions of a goal-relevant stimuli (Summerfield et al., 2011). More broadly, theta often signals both the need for cognitive control and the instigation of that control (Cavanagh & Frank, 2014), but whether that signal is a general alarm or carries specific information is not clear. Here, we examined whether the modulation of theta power by expectation and repetition of a stimulus is dependent on task-relevance of the stimuli. We predicted theta power would differentiate expected and unexpected repetitions for target stimuli, in line with findings from Summerfield and colleagues (2011) but may not show such specificity towards distractor stimuli.

Finally, given the possibility that reduced neural signals of an expected distractor could nevertheless still indicate more efficient stimulus processing (as has been shown in target processing; Kok et al., 2012), we explored the impact of distractor repetition on the neural tuning in the EEG and MEG data. This analysis examined the time course of classification accuracy of stimulus-evoked activity at target and distractor locations as well as investigating whether the altered neural components and activity during distractor repetitions reflected broadened or sharpened neural tuning of distractor features (and therefore potentially reflecting poorer or improved neural representation, respectively). With early evidence suggesting decoding of distractor stimulus features from stimulus-evoked activity are reduced when distractors are more predictable (van Moorselaar et al., 2020; van Moorselaar & Slagter, 2019), we predicted reduced quality of spatial and feature representation after a distractor repeats to the same location.

## Methods

### Participants

Thirty-six volunteers participated in the EEG component of the study. Data sets of six participants were removed from the analysis due to excessive blink trials (>20%) or reaction times greater than two standard deviations from the subject mean resulting in thirty remaining participants (13 female, aged between 20–44 years (mean = 26.27 years; standard deviation = 5.56 years). Seventeen volunteers participated in the MEG component of the study of which eight had also participated in the EEG task. One subject was removed from the analysis because reaction times were greater than two standard deviations from the mean, reducing the total to sixteen (nine female, aged between 19–37 years (mean = 25.0 years; standard deviation = 4.20 years). All participants reported being right-handed, having no history of neurological disease, and having normal or corrected-to-normal vision. They provided written informed consent for being tested according to a protocol approved by the Central University Research Ethics Committee (CUREC). All participants received monetary compensation of £10/h for their time.

### Design

**Fig. 1:**
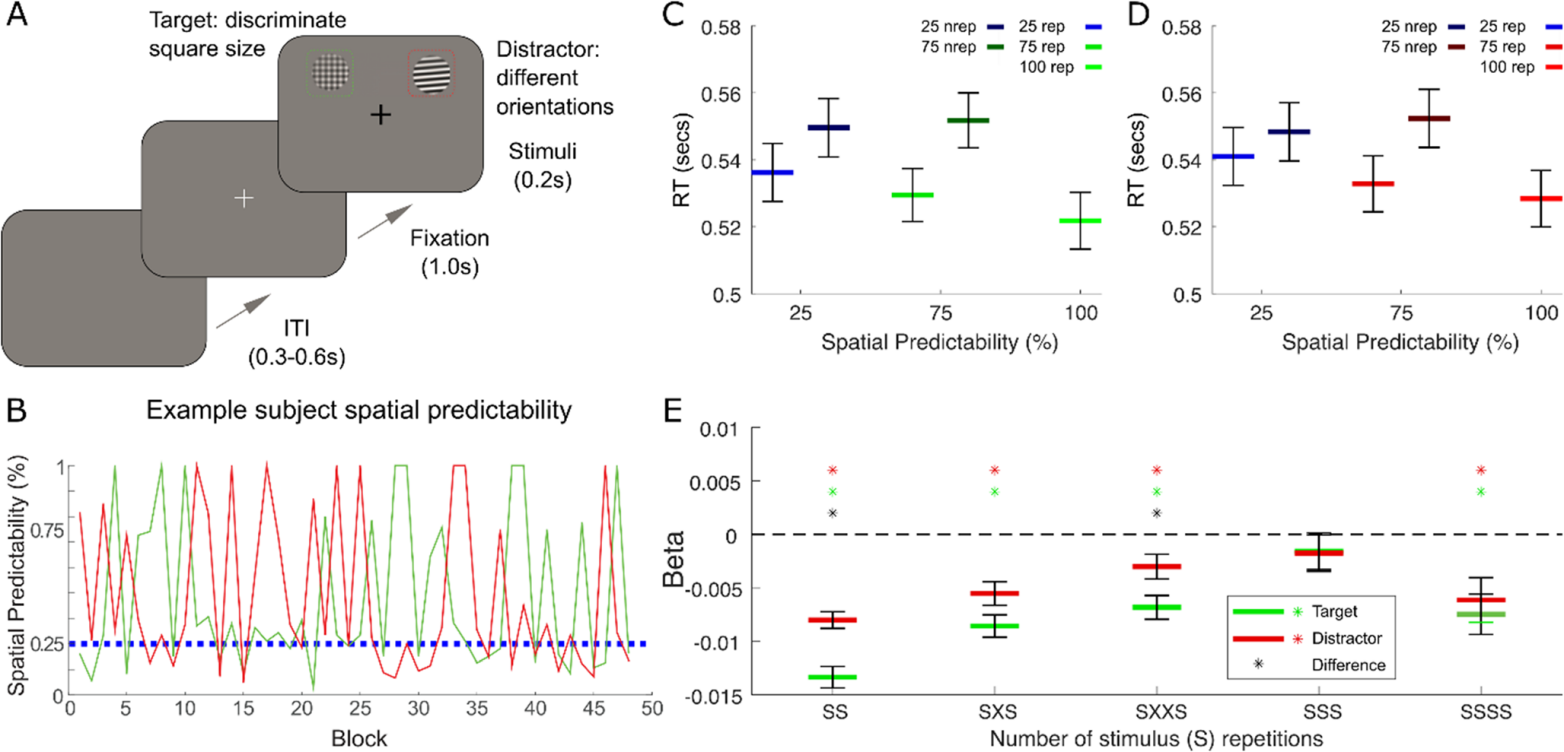
Task schematic, design, and behavioural impact of target and distractor expectation and repetition. **a** Schematic of the experimental task. Participants judged whether target chequered patterns were of high frequency (‘small squares’) or low frequency (‘large squares’). Distractors were gratings oriented in one of 16 angles. Targets and distractors could appear in one of four locations (upper and lower left and right). The target or distractor could appear in each location across a block of 30 trials (±2 trials) with three levels of differing degrees of certainty: 25%, 75%, and 100%. On each trial, participants were required to fixate for 1 second prior to the onset of the stimuli, which appeared for 200 ms. Participants judged whether the target pattern was low/high frequency and responded by right/left button press and received auditory feedback in the form of high and low tones. Then, the fixation cross turned white to mark the intertrial interval. **b** The ongoing spatial predictability of the two stimuli in an example subject test session across the 48 blocks for targets (green) and distractors (red). Blue dashed line marks random spatial predictability at 25%. Note that if the target stimulus occupied a location for a period of time in the 75% and 100% condition, then the spatial uncertainty of the distractor was reduced and therefore its spatial predictability was higher than 25%. Conversely, the same is true for the target when the distractor stimuli was in the higher spatial predictability condition. **c** Mean and standard errors of RTs when the target has a 25% (blue), 75%, and 100% chance of appearing at a particular spatial location, divided according to whether the expected stimulus, target (green) or distractor (red) repeated to the same location (rep: lighter colours) or did not repeat to the same location (nrep: darker colours). **d** Mean and standard errors of RTs when the distractor is the expected stimulus. Format is same as (**c**). Treps and Dreps were independently pooled across the T25 and D25 conditions. Results suggest that reduced RTs with increased expectation were driven by expected stimulus repeats for both targets and distractors. e Regression beta weights (and standard error) reflecting variance explained in RTs by the different numbers of stimulus (S) repetitions of either the target (green) or distractor (red). The model includes single repetitions of expected stimuli (SS), *n*-back repetitions (SXS, SXXS) with intervening trials not filled by the expected stimulus but could be unfilled or filled by the unexpected stimulus (denoted by X), and cumulative repetitions of expected stimuli (SSS, SSSS). Results suggest that target repetitions consistently explained more RT variance than distractor repetitions. For both targets and distractors most RT variance is explained by a single stimulus repetition with increasingly less additive benefit if the repetition was further back in time. Black asterisk (*) denotes significant (*p* < .05) difference between the amount of variance explained between target and distractor conditions, green and red stars indicate significant amount of variance explained by target and distractor conditions relative to zero, respectively. (Colour figure online)

Figure 1 schematically represents the task design and trial sequence. The four-location visual search task required participants to make two-alternative forced-choice target discrimination judgements while the spatial predictability of targets and distractors was implicitly varied across time. The location of the target (T) or distractor (D) was manipulated to repeat with differing degrees of spatial predictability to the same location across blocks of varying trial lengths and series of trials. Spatial predictability for the target or distractor was either 100%, 75% (i.e., the target or distractor had a 75% chance of repeating to the same location or a 25% chance of appearing in any one of the three other locations), or 25% in which the target stimuli had an equal chance of appearing at any one of the four locations while the distractor had an equal chance of appearing at any of the nontarget locations. For each manipulated stimulus (target or distractor), this resulted in three blocked conditions, henceforth referred to as T25, T75, and T100, for the three spatial predictability levels for targets, and analogously, D25, D75, and D100, for distractors. Please note that the conditions T25 and D25 are different labels for the identical control condition in which a given stimulus would appear in a fixed location one out of four times and consequently are pooled in some subsequent analyses.

### Stimuli

Target stimuli consisted of round checkerboard-like patches that were generated by linearly superimposing two orthogonal Gabor patches (i.e., sinusoidal gratings masked by a Gaussian hull; size visual angle = 3.645°; phase = 90°; sigma/spatial constant of Gaussian hull = 25). For the discrimination task, the checkerboard-like targets differed in their “square size”—that is, the spatial frequency of the overlapping Gabors (cycles per pixel: 0.0345 (= small squares) vs. 0.0275 (= big squares). The distractor stimuli were simple oriented Gabor gratings with the same parameters as targets with the exception that, first, they were manipulated at 16 different orientation angles, and, second, they had twice the contrast of target stimuli to elicit larger signals (the behavioral cost of their presence was ascertained in Experiment 1 of (Noonan et al., 2016). Target stimuli could appear in one of four locations, subtending 8.06° of visual angle from a central fixation cross (0.94° visual angle). Distractors could appear in any of the four locations, except the one currently occupied by the target.

### Procedure

In the main task, participants were instructed to discriminate the square size (spatial frequency) of the target stimulus. Each trial started with the fixation period, marked by a black fixation cross on grey background, and lasting 1,000 ms. Next, the stimulus display was presented for 200 ms, containing both the target and distractor. In the response period, participants indicated target differences by pressing the allocated keyboard response buttons “c” or “m.” Response allocations were counterbalanced across participants to avoid systematic differences. Participants were encouraged to ignore the distractors. They received auditory feedback on their response accuracy, via a 50-ms presentation of a high tone (900 Hz) for correct and low tone (500 Hz) for incorrect responses. Lastly, the fixation cross turned light-grey to mark the inter-trial interval, variably lasting 300–600 ms (to reduce the effect of temporal regularity between trials on response times). From there, the trial cycle started anew, marked by a black fixation cross. Participants were encouraged to fixate and minimize their blinks.

Subjects performed 48 blocks, counterbalanced for stimulus locations, target square size, and number of block repetitions (eight blocks per condition), and delivered in a randomly permuted sequence. Block length varied dynamically for each participant (mean = 30; *SD* = 2), so that each participant completed an average of 1,440 ± 96 trials. All trials were delivered in a continuous stream without any explicit breaks between blocks or information about block type. Breaks were scheduled every seven blocks (averaging every nine minutes), creating seven task blocks, in between which participants would receive measures of their reaction time and accuracy to increase task engagement (they were instructed to aim for 90% correct trials). Participants who had not previously participated in a behavioural pilot study were given ca. 3–4 minutes of practice to reduce training effects.

During each break, participants performed a simple distractor localization task, in which the display consisted of only the distractor stimulus that varied in orientation on each trial. The purpose of these intermittent blocks was to experiment with a novel cross-condition decoding method that trains on the single distractor stimulus and applies this training to multiple stimuli (Wolff et al., 2015). Yielding no relevant results, this task added an average of four minutes to each task block and is not further discussed in the present report. Including this task and breaks, the total experimental time was approximately 75 mins.

The experimental script was generated and all stimulus displays delivered dynamically via the Psychophysics Toolbox (Brainard, 1997) in MATLAB (The MathWorks Inc., Natick, MA). In the experiment, for the first 17 subjects, stimuli were presented using a Dell Optiplex 780 computer (with MATLAB version 2011b). However, for the remaining subjects, stimuli were presented using a Dell Optiplex 9020 PC (with MATLAB version 2014a). Both computers projected to a Samsung Sync-Master 2233 monitor (60 Hz refresh rate, 1,680 × 1,060-pixel screen resolution) and were connected to a Dell Optiplex 760 to record the EEG time series. For both computers, we estimated the offset in trigger stamp time and actual stimulus presentation with a photodiode. There was a 6-ms delay between trigger stamp and stimulus presentation for the first computer and a 3-ms delay for the second computer. These respective offsets were corrected for in the subsequent preprocessing steps. In the MEG study, participants were inside a sound-attenuated and magnetically shielded room. For EEG stimuli were presented on the screen at 55 cm (93 cm in the MEG study) distance from the subject and 8 (13) cm lateral to the central fixation cross. For MEG, stimuli were projected from a Panasonic DLP Projector (PT-D7700E) with a spatial resolution of 1,024 × 768 pixels and a refresh rate of 60 Hz. Viewing distance was constrained by the MEG scanner to be 93 cm.

EEG time-series data were recorded using a NeuroScan SynAmps RT amplifier and Scan 4.5 software (Compumedics Neuroscan, Charlotte, NC), measuring from 60 individual scalp Ag/AgCl surface electrodes mounted on an elastic cap (EasyCap, Herrsching, Germany) according to the international 10–20 system. Electro-oculograms were recorded via two bipolar channels from supraorbital and infraorbital right eye electrodes (vertical EOG), and from the electrodes placed at external canthi of the eyes (horizontal EOG). Data were referenced online to the right mastoid (TP10) and rereferenced offline to the average of the left and right mastoid electrodes. The anterior frontal midline electrode was used as a ground.

MEG data were acquired on a 306-channel VectorView system (Elekta Neuromag) with two orthogonal planar gradiometers and one magnetometer at each of 102 locations allocated in a helmet surrounding the top of the scalp, situated within a magnetically shielded room. During acquisition, a band-pass filter of 0.03–330 Hz was applied and the head position continuously monitored using four head position indicator (HPI) coils attached to the scalp. Before data acquisition, the HPI coil locations, three anatomical fiducial locations—the nasion and left and right preauricular points—and head points across the scalp were digitized using a Polhemus Isotrak II. Furthermore, to detect eye movements and heartbeat, we measured the horizontal and vertical electro-oculogram and electro-cardiogram via electrodes attached to the eyes and forearm. Before starting the MEG task, an EyeLink 1000 eye-tracking system was calibrated to an individual’s head position and eye movements were recorded continuously.

### Preprocessing: EEG

EEG-recordings were re-referenced offline to the average of the mastoid electrodes. The data were resampled to 250 Hz with 16-bit precision and band pass filtered (0.05–40 Hz) using EEGLAB (Delorme & Makeig, 2004). Obtained triggers from the onset of epochs were realigned to account for timing lag between the trigger and stimulus onset. Time-series data were epoched using the digital triggers sent by the computer executing the MATLAB script to the computer recording the EEG-data. For the event-related potential (ERP) analyses, the data were epoched between −0.25 s and +0.4 s relative to the stimulus onset. Trials contaminated by excessive artifacts (e.g., eye blinks, saccades) were identified by visual inspection and removed. An automatic criterion was also used to detect remaining artifacts on any channels included in the analysis (±50 mV). If this resulted in <25 remaining trials in any condition, the participant was excluded from that analysis (one instance described below). Data were baseline-corrected with respect to the average voltage at −250 and 0 ms.

For time frequency (TFQ) analyses, the data were epoched between −0.5 s and +1.0 s relative to the stimulus onset and decomposed between 4–8 Hz (theta) and 8–12 Hz (alpha) at increments of 1 Hz using FieldTrip (Oostenveld et al., 2011). The analyses were conducted using a Hann multitaper method with three cycles per taper. Trials contaminated by excessive artifacts as identified in the shorter epoch were removed, in addition to trials with amplitude ±50 mV or power ±500. No baseline was applied as the block design meant that the cueing effects might manifest at any point within the trial. Power was log-transformed and averaged across this theta range.

### Preprocessing: MEG

All MEG data were analyzed using the FieldTrip toolbox (Oostenveld et al., 2011) and custom-written scripts for MATLAB (The MathWorks & MATLAB, 2021a). First, bad channels were identified by visual inspection. External noise was removed from the data using MaxMove (Elekta Neuromag) software and applying the signal-space separation method (SSS) with its temporal extension (ST). Continuous movement compensation, as indicated by the HPI coils, were applied and each individual’s data transformed to the coordinate frame of their first scanning block. Before conversion to the SPM format, the continuous data were bandpass-filtered at 0.1–500 Hz, down-sampled to 500 samples per second, then epoched with respect to stimulus onset in a time window of −0.5 to 0.75 s. Time points in which artifacts resulting from muscles, blinks, saccades, and signal dropout occurred were marked by visually inspecting all trials. In addition, we used Independent Component Analysis (ICA) to remove eye-motion and heart rate artefacts.

### Data analyses

#### Reaction times and accuracy

Analyses were performed in MATLAB (R2021a) and SPSS (Version 27). We calculated median reaction time from stimulus onset (RT) and mean response accuracy for each of the three conditions for targets and distractors separately. We explored how the effects of expected repetition interacted with spatial predictability by comparing repetition (rep) and nonrepetition (nrep) trials across conditions. Initially excluding the fully predicted condition we performed two 2 (spatial predictability: 25, 75) × 2 (repetition: rep, nrep) ANOVAs for target and distractor stimuli separately. We also investigated the performance benefits of repetitions under increasing predictably, analyzing repeat trials across all spatial predictability conditions for targets and distractor separately (one-way ANOVA spatial predictability: 25, 75, 100). Trep trials in T25 and D25, and Drep trials in T25 and D25 were independently pooled. Note, for the behavioural RT analysis, the exclusion criteria for single trials within participants were as follows: Trials with deviant latencies three times the median absolute deviation (Leys et al., 2013), error trials, trials after a break and the first four trials in each new spatial predictability block as new expectations would be expected to not be fully established in these trials. This latter criterion was applied to any later analysis (behavioural or EEG) that compared across spatial predictably blocks. Accuracy had the same exclusion criterion with the exception of error trials.

To assess the longer term impact of trial history repetitions, we collapsed across all spatial predictability conditions and estimated the residuals of log-transformed RT after accounting for the (i) the target location (upper or lower quadrant and left or right hemifield), (ii) targets and distractors in the same hemifield, (iii) target identity (small or large squares), (iv) repetition of target identity. RT outliers, error trials and postbreak trials were excluded before residual RT was calculated. We regressed the residuals of RT against five categorical factors of stimulus (target or distractor) repetition. The terms included in the model characterized single trial repetitions (TT), *n*-back (TXT & TXXT, with X exclusively not a T, but could be a D), and DXD and DXXD (with X exclusively not a D, but could be a T) and cumulative repetitions of targets and distractors (TTT, TTTT, and DDD, DDDD). Beta estimates were compared in a 2 (stimulus type; target and distractor) × 5 (repetition factor; SS, SXS, SXXS, SSS, SSSS) repeated-measures ANOVA and *t* tests against zero and between target and distractors.

#### Trial-wise target and distractor stimulus repetition effects

All EEG data were analyzed using the FieldTrip toolbox (Oostenveld et al., 2011) and custom-written scripts for MATLAB (The MathWorks & MATLAB, 2021a). First, we examined ERP waveforms for channels that were contralateral (contraT) and ipsilateral (ipsiT) to target position. Any trial in which the target appeared in the right hemifield was flipped into the left hemifield. Only trials in which both stimuli were presented on the same vertical plane were analyzed, meaning that contraT is equivalent to ipsilateral to distractor (ipsilaD) and ipsiT is equivalent to contralateral to distractor (contraD). Upper and lower visual fields were analyzed separately as early components are known to differ relative to stimulus position on different sides of the horizontal meridian (Di Russo et al., 2002). All lateralized posterior and occipital electrodes were included in the analysis (P2, P4, P6, P8, PO4, O2, PO8, P1, P3, P5, P7, PO3, O1, PO7). Specifically, we examined the neural mechanisms common to any repetition of a target or distractor stimulus. Collapsing across the three Spatial Predictability conditions we compared any target or distractor repetition (Trep, Drep) with trials in which there was no repetition (Tnrep, Dnrep). Again, only lateralized target/distractor configurations were included, and upper and lower lateralization are plotted separately. Trials at the beginning of each spatial predictability block were not removed for this analysis. We used an a priori time windows interest centred over the early P1 visual components. Waveforms for time of interest analyses included all trials to ensure subsequent contrasts were orthogonal from the derived peaks. The P1 was defined as the maximal peak of the waveform prior to the N1 (itself identified based on the global peak negativity). We restrict this analysis to stimuli presented lateralized in the upper hemifields (to avoid the positive-going C1 blending into the later P1 in lower hemifield stimulus configurations). Voltages were extracted from each subject from a 20-ms window centred on the group average peak value (134–154 ms). In order to directly compare P1 activity across the two stimulus types, we conducted a complementary analysis where we removed trials in which the other stimulus repeated to the same location. For example, in the Trep and Tnrep conditions, we removed trials in which the distractor also happened to repeat. The equivalent step was also performed in the Drep and Dnrep conditions. We subjected the average ERP activity across the P1 to a 2 (stimulus type; target, distractor) × 2 (stimulus repetition; repeat, nonrepeat) repeated-measures ANOVA. We also repeated this analysis for ERP activity averaged over a 20-ms window centred over the average N1 peak and the N2pc time window defined by the cluster in the lateralized waveform that showed significant differences between Drep and Dnrep (see below).

To complement these focused analyses, we also conducted group level cluster corrected nonparametric permutation-based tests (*p* < .05) that estimated statistical differences between conditions across time (Maris & Oostenveld, 2007). This method involves estimating the *t*-statistic across participants for a contrast of interest at each time point, defining observed clusters of consecutive above-threshold time points, and calculating the cluster mass (by summing all *t* values in an above-threshold cluster). Next, condition labels were randomly shuffled within participants (sign-flip for contrasts against zero), and the largest cluster mass produced by chance was extracted. This permutation step was performed 10,000 times to estimate the null distribution. The probability of the observed group-level cluster against chance was then derived as the rank order of the observed cluster relative to the null distribution (Myers et al., 2014). Finally, to assess spatiotemporal differences in later ERP components, all channels were plotted as spatial topographies using the field trip toolbox. Epoched data are subjected to the function “Ft_timelockstatistics,” which computes Monte Carlo cluster-corrected statistics across space (with at least two neighbouring channels) and time (latency 0–0.4 s), taking the maximum sum of the *t* values within every cluster (*p* < .05, permutations = 1,000). We examined two contrasts (1) target repetition vs. nonrepetition, (2) distractor repetition vs. nonrepetition and cluster corrected across space and time.

We also calculated lateralized waveforms (contraT–ipsiT ERP activity) for repeat and nonrepeat trials for target and distractor stimuli and subjected them to the same group level cluster corrected nonparametric permutation-based tests (*p* < .05) that estimated statistical differences between repeat and nonrepeat trials across time.

#### Effects of higher order expectation of target and distractor stimuli on voltage amplitudes

To investigate the effect of higher order expectation on local stimulus processing, we regressed the EEG amplitude with the varying trial-wise estimates of expectation of target or distractor location derived from a reinforcement learning model described in the Supplementary Material. Importantly, we sought expectation independent from repetitions and so included in the regression analysis the repetition of target or distractor at a particular location. Resulting beta values were normalized by dividing each time point, for each subject and channel by the maximum beta value identified for each trial. Mean beta values of target and distractor expectation for contraT and ipsiT locations were extracted from a 20 s time window centred over the peak P1 amplitude as identified in the raw EEG waveform (see above). The beta values for the four conditions were subjected to independent one-sample *t* tests against zero. We also compared the mean beta coefficients for targets and distractors against zero for all time points and all channels before comparing beta coefficients of targets against distractors. All trials were included regardless of upper and lower locations. Spatial topographies of the beta coefficients are estimated as described above, plotted, and cluster corrected statistics were applied across space and time (*p* < .05).

#### Effects of higher order expectation of target and distractor stimuli on frontocentral theta power

To investigate the effect of higher order expectation on frontocentral theta power, we regressed logged theta power (from C2, C1, C4, C3, CP4, CP3, CPz, Cz, FC3, FC1, FCz, FC4, FC2 channels) with the varying trial-wise estimates of expectation of target or distractor location. At the group level, we compared the mean beta coefficients for targets and distractors against zero across time in cluster corrected nonparametric permutation-based one-sample *t* tests using the methods described above, and against each other with equivalent paired-sample *t* tests.

#### Alpha power after expected and unexpected target and distractor repetitions

Next, we investigated stimulus evoked alpha power separately for target and distractor condition as a function of repetition (rep and nrep) and spatial predictability (25 and 75). Averaged log transformed alpha power (from P2, P4, P6, P8, PO4, O2, PO8, P1, P3, P5, P7, PO3, O1, PO7) in each condition was calculated relative to each stimulus and pooled across stimuli in any location or configuration. Cluster corrected nonparametric permutation-based tests (*p* < .05) compared alpha power across time between stimulus repeat and non-repeat trials for contralateral and ipsilateral alpha separately.

#### Decoding of distractor stimulus features in MEG and EEG data

Finally, we examined whether distractor repetition altered perceptual representations of key features of the distractor such as location or orientation. Past work has demonstrated that increased target expectations were associated with decreased neural activity but increased feature decoding (Kok et al., 2012). We examined this latter possibility in the case of distractor expectation in the MEG and EEG data. First, we decoded distractor spatial location by applying a principle component analysis (PCA) to the time series data over all trials and channels in the MEG data. For decoding, we used the first 60 PCA components. For each time point from −100 to +400 ms relative to stimulus onset, we took the surrounding samples at ±40ms, de-meaned this window and made five 16-ms bins. Within each bin, the data were averaged over time. For decoding, we trained and tested on separate data sets. Trials from the localizer task were used for training and those of the main task were used for testing. For each time point we used the previously computed averaged time bins and computed for each test trial the standardized Euclidean distance to the average of the training set between each location. Because of standardization via PCA, this is equivalent to taking the Mahalanobis distance. To maximize the congruence to MEG decoding, we performed identical decoding-specific processing steps in the EEG data and extracted the same amount of 60 PCA components that explained the most variance for location and orientation decoding. To compare distractor repetition with nonrepetition trials, we calculated how often the shortest distance accurately predicted the actual distractor location for trials of each type subtracted chance-level accuracy (0.25), and smoothed individual subjects’ time series with the neighbouring ±10 ms. To statistically test whether nonrepetition trials had higher decodability, we computed a cluster-based permutation test from 50,000 equal sized random trial selections using data from all subjects (Maris & Oostenveld, 2007). Clusters exceeding a family-wise error (FWE)-corrected alpha of 0.05 were deemed significant.

Next, we decoded distractor orientation, which varied randomly on a trial-wise basis. Because of this variation very few trials were identical, which is a prerequisite for decoding. To increase training and test sets we grouped trials into eight different orientations and used a 10-fold cross-classification over the whole data set. Standardized Euclidean distance was computed between each trial and the average of the training set for each of the eight orientations. Because similar orientations should result in shorter distances, the difference between similar to dissimilar orientations indicates tuning to orientations, here expressed as the fit to a cosine function. This cosine fit as a measure of tuning to orientations was estimated for each of the time steps, resulting in a 2D heat map of tuning over time. We subjected the overall tuning curves over time to a cluster-based permutation test procedure with 50,000 random resamples that tested the tuning against chance. Clusters exceeding a, FWE-corrected *p* < .05 were deemed significant. Using the overall tuning to distractor orientations, we tested whether distractor and target repetitions had an effect on decodability of the distractor orientation by taking the average peak decoding per subject for repetition and nonrepetition trials and subjecting these individual subjects means to two independent paired-samples *t* tests: Drep vs. Dnrep and Trep vs. Tnrep.

## Results

### Repetition and expectation of targets and distractors improve measures of performance

In a task designed to implicitly manipulate stimulus repetition independently of spatial expectation for task relevant and irrelevant information, we show that stimulus repetition and expectation of both targets and distractors reduce RTs (Fig. 1c–d). Two independent repeated-measures ANOVAs confirmed this pattern for the low (25%) and high (75%) spatial predictability conditions. RTs are reduced when targets repeat to the same location, Repetition: *F*(1, 29) = 102.05, *p* < .001, an effect that was additionally dependent on spatial predictability, Repetition × Spatial Predictability: *F*(1, 29) = 5.89, *p* = .022. The same effects are also evident for distractor processing, with repeated distractors reducing RT to target discrimination, Repetition: *F*(1, 29) = 35.50, *p* < .001, an effect amplified by increasing predictability of the distractor stimulus, Repetition × Spatial Predictability: *F*(1, 29) = 7.85, *p* = .009. Furthermore, RTs are reduced with increasing spatial predictability for both targets and distractors (one-way ANOVAs of 25%, 75%, and 100% repetition trials) *F*(2, 58) = 9.60, *p* < .001, and *F*(2, 58) = 4.84, *p* = .012, respectively.

Complementary analyses in the accuracy data (Fig. S1A–B) revealed some conserved but weaker effects for target repetition. While no effects were significant in the 2 (repetition) × 3 (spatial predictability) ANOVA for either target or distractor stimuli (all *p*s > .115), accuracy to report the target category in repetition trials did increase with increasing predictability (one-way ANOVA of target repeat trials for 25%, 75%, and 100%), *F*(2, 58) = 3.56, *p* = .035. By contrast, there were no significant effects in the equivalent distractor analysis, *F*(2, 58) = 1.49, *p* = .235. Importantly, there was no RT–accuracy trade-off in the distractor condition (D25 vs. D100 repetition trials), *t*(29) = 0.701, *p* = .489. These patterns in RT and accuracy were replicated in the behavioural data collected alongside the MEG data (see Supplementary Material and Fig. S1C–F).

Next, we assessed the longer term impact of trial history repetitions, for targets and distractors, collapsed across all spatial predictability conditions and independent of a number of potentially confounding variables (see Methods, Fig. 1e). Comparison of regression-derived beta estimates in a repeated-measures ANOVA of Stimulus Type and Repetition Factor suggests target repetitions explained broadly more variance in RTs than distractors, Stimulus Type: *F*(1, 28) = 16.07, *p* < .001, and different repetition factors account for differential RT variance, Repetition Factor: *F*(4, 112) = 9.94, *p* < .001, with most variance being explained by the single most recent stimulus repetition, post hoc *t* test against zero, TT: *t*(29) = −13.08, *p* < .001; DD: *t*(29) = −10.10, *p* < .001. Indeed, there was increasingly less additive benefit for repetitions that occurred further back in time, TXT: *t*(29) = −8.08, *p* < .001; DXD: *t*(29) = −4.91, *p* < .001; TXXT: *t*(29) = −6.04, *p* < .001; DXXD: *t*(29) = −2.55, *p* = .016. Additional cumulative repetitions only significantly affected RT when there are four consecutive repetitions of either target, TTT: *t*(29) = −0.90, *p* = .376; TTTT: *t*(29) = −3.89, *p* = .001, and distractor, DDD: *t*(29) = −1.01, *p* = .320; DDDD: *t*(29) = −291, *p* = .007.

### Trial-wise stimulus repetition effects differentiate target and distractor processing

**Fig. 2:**
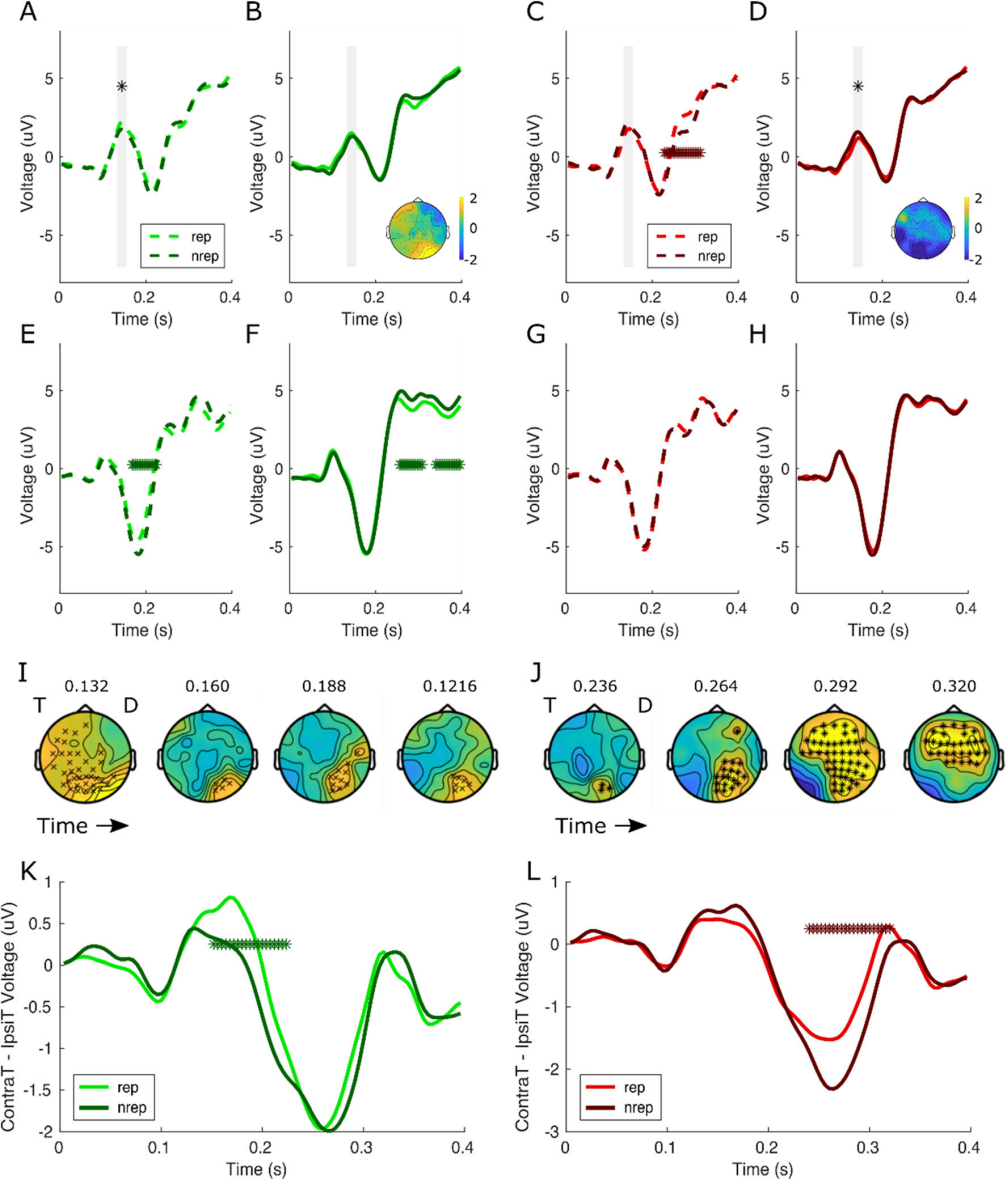
Distractor repetitions diminish P1 amplitude and N2PC. ERP waveforms average from occipital and posterior electrodes for target (green) and distractor (red) repetition (rep: light colours) and nonrepeat (nrep: dark colors) plotted separately for sensors contralateral to target (contraT: dashed lines) and ipsilateral to target (ipsiT: solid lines) when stimuli were presented in the upper (**a***–***d**) or lower (**e**–**h**) visual field. Significance bar indicates cluster-corrected (*p* < .05) *t* tests between repeat and stimulus-specific nonrepeat trials. Gray panels represent a 20-ms window centered over the peak of the P1 components only readily identifiable in the upper visual field due to overlap with C1 in the lower field. Asterisks indicate significant differences between repeat and nonrepeat conditions using nonparametric permutation-based correction. Repeated targets increase the P1 amplitude contralateral to the target stimuli relative to nonrepeat trials. By contrast, distractor repeat trials result in smaller P1 amplitude contralateral to the distractor stimuli relative to nonrepeat trials. Insets: Topographies of the P1 repetition effects (repetition vs. nonrepetition) averaged between 0.132 and 0.152 s are illustrated to show the relative lateralized posterior distribution. **i***–***j** Spatially and temporally cluster-corrected topographies for target and distractor for repetition minus nonrepetition trials, respectively. Target repetition manifests in a single positive cluster between 0.120 *and* 0.228 s. By contrast, distractor repetition manifests in a single positive cluster from 0.236 *to* 0.336 s. T and D represent the location of the target and distractor, respectively. Separate significant clusters for target and distractor are denoted by ^x^ and * symbols, respectively. **k***–***l** Lateralized components were isolated for target and distractor (respectively) by subtracting ipsilateral waveforms from contralateral waveforms from the respected expected stimulus for both stimulus repeat (lighter colours) and nonrepeat (darker colours) trials. Repeated distractors result in a reduced N2pc component, whereas repeated targets are associated with an enhanced lateralized component at an earlier timepoint. (Colour figure online)

The behavioural results suggest that both stimulus repetition and increased expectation of both targets and distractors improve task performance. Turning to the neural data, we therefore asked whether the similar behavioural profiles would be underwritten by related or distinctive neural mechanisms. First we examined the impact of a single repetition of either target or distractor on ERP components, as behaviourally this factor had the largest impact on RT. Pooling over objective spatial predictability (Fig. 2) we show that a single trial repetition of a distractor decreases P1 amplitude contralateral to distractor location (Fig. 2d), *t*(29) = −2.28, *p* = .03, and is complemented by an enhancement of P1 amplitude contralateral to target location when targets repeat to the same location (Fig. 2a), *t*(29) = 2.17, *p* = .039. Direct comparison between targets and distractor ERP activity at the P1 time window revealed a significant difference, Stimulus Type: *F*(1, 29) = 21.74, *p* < .001, a difference that was modulated by whether the stimulus repeated or not, Stimulus Type × Repetition: *F*(1, 29) 8.92, *p* = .006. However, there was no independent effect of Repetition, *F*(1, 29) = 0.54, *p* = .479. Repeating the analysis for the N1 component showed a similar difference between target and distractor activity overall, Stimulus Type: *F*(1, 29) = 18.80, *p* < .001, but no other significant effects, Repetition: *F*(1, 29) = 0.07, *p* = .790, Stimulus Type × Repetition: *F*(1, 29) = 0.03, *p* = .862.

Spatial topographies confirm relative spatial distinctions between target and distractor repetition effects and their confinement to posterior electrodes at the P1 time window (Fig. 2 insets). However, neural differences distinguish between repetitions of target and distractor stimuli later in time. For targets we see reduced amplitude contralateral to target in the lower visual field channels between 0.168 and 0.224 s (Fig. 2e, *p* = .010) corresponding to the N1 and ipsilateral to target in two clusters between 0.256 and 0.308 s and 0.340 and 0.396 s (Fig. 2f, *p* = .011 and *p* = .02, respectively). By contrast, after distractor repeats, signals are enhanced contralateral to target when in upper channels between 0.228 and 0.312 s (Fig. 2c, *p* < .001).

Next, we investigated the spatial-temporal topography of the repetition effects on amplitude and again demonstrated differential processing of the two stimulus categories. Target repetition manifested in a single positive cluster from 0.120–0.228 s (Fig. 2i, *p* = .031). Beginning before the time of the P1, this cluster begins relatively nonlateralized, growing anteriorly into a large posteriocentral cluster. By the time of the P1, this cluster has refocused on posterior electrodes contralateral to the target. By contrast, distractor repetition manifested in a single positive cluster at later time points from 0.236–0.336 s (Fig. 2j, *p* = .001). This cluster begins at posterior electrodes contralateral to the target and progresses anteriorly with time. This neural signal likely includes the distractor repetition effects seen in Fig. 2c.

Lateralization of the waveforms also suggests differences between target and distractor repetition. Repetition of the target amplifies contralateral signals relative to ipsilateral signals between 0.152 and 0.224 s (Fig. 2k, *p* = .001) at the P1 time window. By contrast, repetition of a distractor reduces the N2pc, potentially reflecting less attentional capture by the distractor (Fig. 2l, 0.240–0.320 s, *p* < .001). Again, direct comparison between ERP activity at the N2pc time window for targets and distractors revealed a significant main effect of Stimulus Type: *F*(1, 29) = 33.48, *p* < .001, and Repetition: *F*(1, 29) = 26.92, *p* = < .001, with reduced lateralization for both targets and distractors after either stimulus repeated to the same location. The two factors did not interact, *F*(1, 29) = 0.78, *p* = .384.

Notably, the data from the current experiment also lends itself to be analyzed in the same way as described in our previous study (Noonan et al., 2016). For completeness, we repeat the past analyses and show that our past distractor effects in the P1 and N2PC replicated in this new sample (Fig. S2).

### Distinct time courses of target and distractor processing in markers of neural expectation

Having shown that the repetition of a target and distractor stimulus produces different effects on the ongoing ERP time courses, we next investigated whether the higher order expectation of stimulus repeating to a particular location, as shaped by stimulus history effects, similarly differentiated the neural signals to target and distractor stimuli. To do so, we first estimated trial-wise expectation using a reinforcement learning model. Model construction, tests and comparison are detailed in Supplementary Materials (Fig. S3A–C) through which we replicated behavioural findings by simulating RT data from the model fits (Fig. S3D–E). We regressed the parametric expectation for a target or distractor at each location on every trial to the corresponding ERP amplitude. Our model included covariates of single-trial repetitions meaning that expectation values are independent of repetition effects. When these are included in the model and we focus a priori on the P1 time window, there is no significant relationship between P1 amplitude and expectation (Fig. S4), Target contraT: *t*(29) = 0.66, *p* = .512; Distractor CT: *t*(29) = 0.13, *p* = .897; Target ipsiT: *t*(29) = 1.55, *p* = .132; Distractor ipsiT: *t*(29) = −0.69, *p* = .496. This indicates these early effects are driven predominantly by the expectation of a stimulus repetition and do not reflect more subtle higher order expectations.

**Fig. 3:**
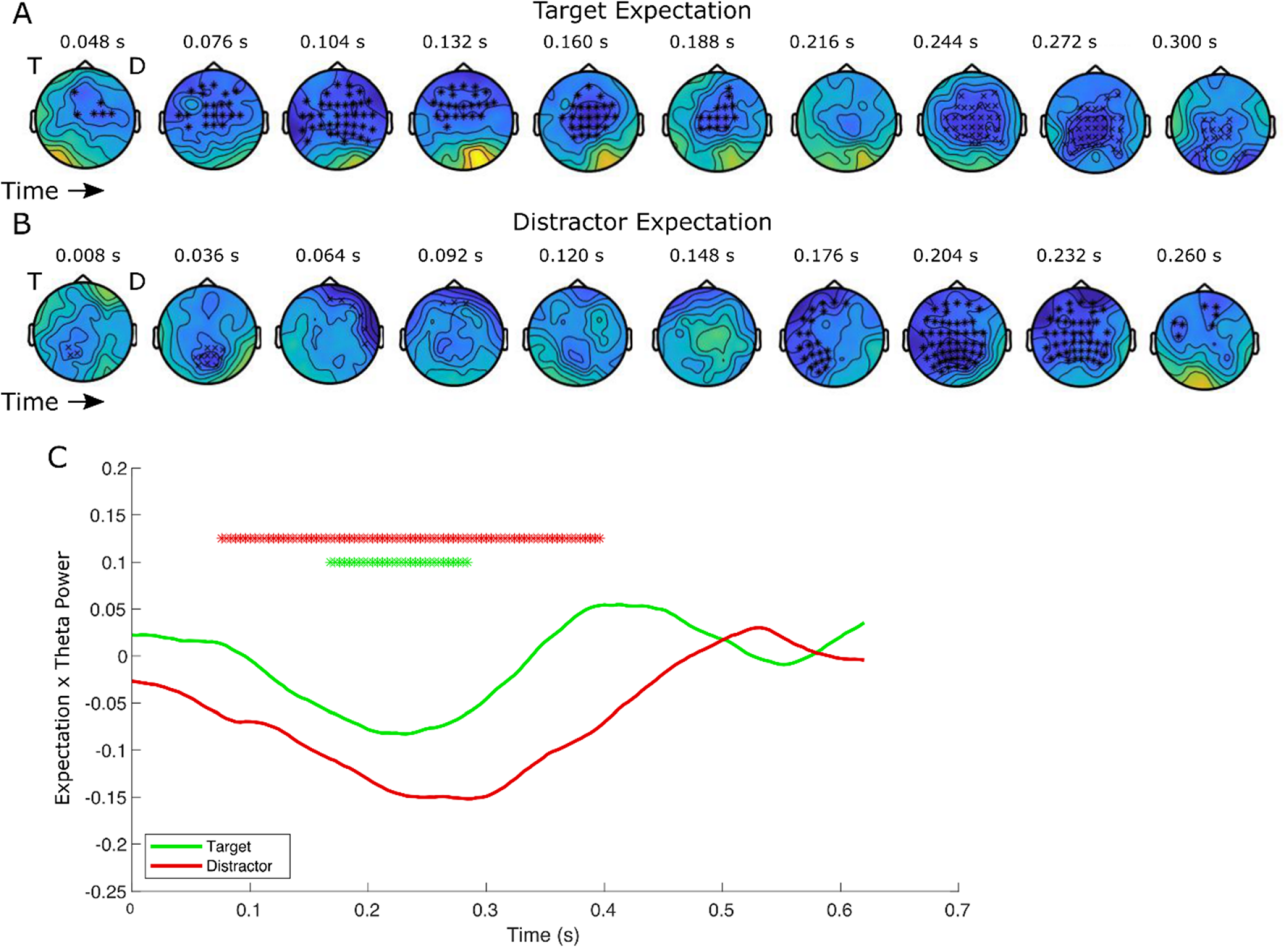
Higher order expectations impact later frontocentral activity and theta. ERP amplitude correlates with target (**a**) and distractor (**b**) expectations (as derived from the learning model, with stimulus repetition regressed out of the variance) in distinct frontal temporal topographies. Target expectation is significantly negatively correlated with amplitude in two clusters from 0.048 *to* 0.208 s (*p* = .009) and 0.22 *to* 0.312 s (*p* = .018). While increased distractor expectation at particular locations is negatively correlated in two cluster 0.008–0.112 s (*p* = .04) and between 0.164–0.280 s (*p* = .005). T and D represent the location of the target and distractor, respectively. The significant clusters for target and distractor are denoted by ^x^ and * symbols. c Frontocentral theta power correlates with target (green) and distractor (red) expectations. Frontocentral theta power decreases with increasing target and distractor expectation in single clusters respectively (target 0.168–0.284 s, *p* = .048 and distractor 0.08–0.396 s, *p* < .001). Asterisks indicate significant differences relative to zero using nonparametric permutation-based correction. (Colour figure online)

By contrast, we observe distinct higher order expectation effects for distractors and targets in more frontocentral signals (Fig. 3). Target expectation is significantly negatively correlated with ERP amplitude in two clusters (Fig. 3a). Firstly, from 0.048–0.208 s (*p* = .014), ERP amplitude decreases with increasing expectation in a relatively sparse central distribution which intensifies over time into frontal channels. Then, between 0.22 and 0.308 s (*p* = .030) the second large frontocentral cluster progresses posteriorally. By contrast, increased distractor expectation (Fig. 3b) at a particular spatial location is negatively correlated with ERP amplitude between 0.008 and 0.112 s (*p* = .037) and again between 0.164 and 0.280 s (*p* = .004). This pattern begins most posteriorly, before rapidly shifting to frontal electrodes and then advancing back posteriorly and broadly over the whole scalp. The P1 time window sits between these two clusters.

Finally, we investigated the expectation of targets and distractors in frontocentral theta power. Frontocentral theta has previously been linked with target expectation, with Summerfield et al. (2011) showing that expected target repetition is associated with reduced theta power relative to trials in which the target was expected to repeat but did not. Here we first tested whether frontocentral theta power was differentially associated with target or distractor expectation. We regressed log-transformed theta power against expectation of targets or distractors in each location on every trial. Note critically that stimulus repetition was regressed out of the variance in theta power. We observe that frontocentral theta power decreases with increasing distractor expectation in a cluster between 0.08 and 0.396 s (*p* = .001). Target expectation is similarly associated with reduced theta but in a much shorter, less pronounced cluster between 0.168 and 0.284 s (*p* = .048). Then, in a complementary analysis, we examined whether theta power distinguished between expected and unexpected repetition differentially for targets and distractors. We compared log-transformed frontocentral theta power across repetition and nonrepetition trials in the high (75%) and low (25%) spatial predictability blocks. This analysis, which was more directly comparable to Summerfield and colleagues, revealed that expected repeats of targets and distractors to the same location were both associated with reduced theta power (Fig. S5A, T75 Trep vs. Tnrep, 0.220–0.376 s, *p* = .254; Fig. S5B, D75 Drep vs. Dnrep, 0.220–0.388 s, *p* = .0352). However, unlike unexpected target repeats (Fig. S5C, TD25 Trep vs. Tnrep, no significant cluster, *p* = .108), unexpected distractor repeats also reduced theta power (Fig. S5D, TD25 Drep vs. Dnrep, 0.100–0.312 s, *p* = .224). This implies that theta distinguishes between repetitions of task-relevant stimuli but not task-irrelevant information.

### Dissociable alpha modulation after expected and unexpected target and distractor repetitions

**Fig. 4:**
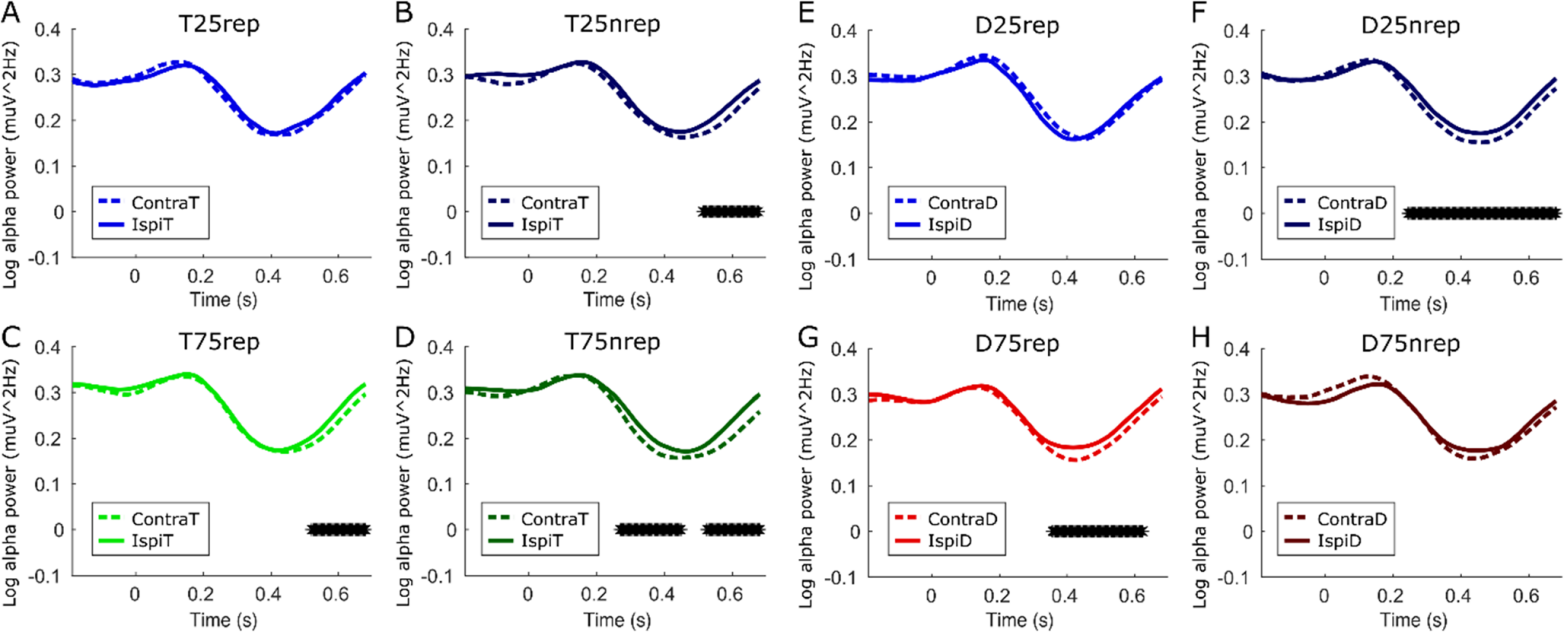
Lateralized stimulus evoked alpha power differentially modulated by expected and unexpected target and distractor repetitions. Log alpha power plotted contralateral (dashed lines) and ipsilateral (solid lines) to stimuli for target (**a**-**d**) and distractor (**e**-**h**) repeated and non repeated conditions. Posterior electrodes contralateral to a target stimulus showed reduced alpha power, relative to ipsilateral electrodes, when expectations of target repetition were fulfilled across the two spatial predictability conditions (T25nrep and T75rep). Alpha power was also reduced contralateral to targets that appear at an unexpected location when expectations were violated (i.e., when a target did not repeat to the same location in the high spatial predictability condition; T75nrep). By contrast, alpha power only showed this lateralization pattern to distractor locations when expectations were fulfilled (D25nrep and D75rep) with reduced alpha over channels contralateral to the location of the distractor. (Colour figure online)

Next, we examined the lateralization of alpha power to the target or distractor location as a function of repetition and spatial predictability (Fig. 4). The results suggested that when targets fulfilled expectations by repeating in a low repeat condition or repeating in a high probability of repeat condition, alpha was reduced over channels contralateral to the location of target (T25nrep *p* = .0476, T75rep *p* = .019). However, alpha also distinguished between the other target conditions in which expectations are violated. Specifically, targets that repeated in a low repeat condition showed no alpha lateralization (T25rep *p* = .999). By contrast, there was significant target lateralization when targets did not appear at an expected location in the high repeat condition and instead appeared at a new location, with reduced alpha over channels contralateral to the new target location (T75nrep two clusters *p* = .0376, *p* = .0474).

When distractors fulfilled expectations by not repeating in a low repeat condition (D25nrep) or repeating in a high probability of repeat condition (D75rep) there was increased lateralization of alpha, with reduced alpha over channels contralateral to the location of the distractor (D25nrep *p* < .001, D75rep *p* = .002). By contrast, when the distractor violated expectations by repeating in a condition with few repeats (D25rep) or not repeating in a condition when it was expected to (D75nrep), there was significantly less lateralization (D25rep *p* = .99, D75nrep *p* = .249), potentially suggesting stimulus evoked increased alpha over contralateral channels acted to suppress the distracting influence of the distractor.

### Decoding of stimulus location and features: Weaker representation of distractor features after distractor repetition

**Fig. 5:**
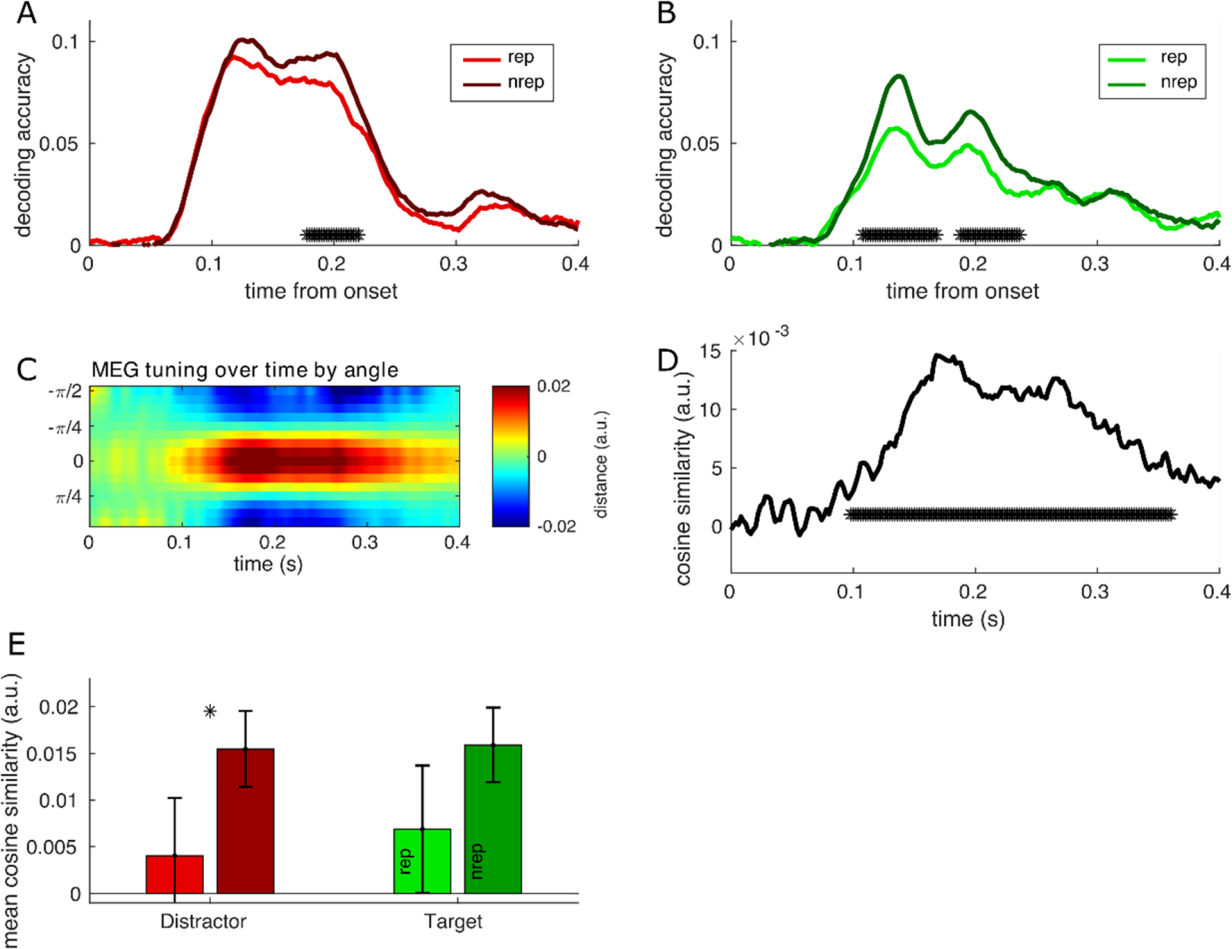
Representation of distractor location and stimulus features diminished by distractor repetition but not target repetition. Accuracy of decoding distractor (**a**) and target (**b**) spatial location is reduced after a single trial repetition (lighter colour) relative to a nonrepetition trial (darker colour). Asterisks denote significant cluster-based permutation tests. Differences in distractor location decoding extend between 0.168 and 0.222 s while target repetition effects are significantly different between 0.116 and 0.160 s. **c** Heat maps show mean standardized Euclidean distance between trials relative to the distractor orientation angle and represents a tuning curve to the distractor angle over time. Hot/red areas reflect higher similarity for similar angles, while cold/blue areas represent lower similarity in dissimilar angles. Higher tuning to distractor angle is visible after 100 ms. **d** Comparison of the tuning curve to a cosine function averaged across participants and representing feature encoding across time. Asterisks denote significant cluster-based permutation tests of subjects’ tuning curves and show significant stimulus features encoding with a peak between 200 and 300 ms. **e** Mean and standard error of the cosine similarity extracted from the peak tuning window for target and distractor repetitions and nonrepetitions. Results suggest distractor repetition diminishes tuning to distractor orientation, but target repetition does not reduce distractor orientation tuning. (Colour figure online)

The distinct effect of target and distractor repetition on ERP components, as well as the distinguishable time courses in ongoing EEG amplitude and theta power, suggests dissociable underlying neural mechanisms support the similar improved behaviour we see after target and distractor repetitions and increased expectation of either stimulus. In particular, we see reduced amplitude after distractor repetitions (P1 and N2PC) and increased expectation of a distractor. However, these analyses cannot tell us whether this reduced activity translates to an effective neural suppression of the distractor. To test this, we investigated whether a single repetition of a stimulus location affected the neural representation of that stimulus in the MEG data and EEG. Examining the neural representations of target and distracter separately, we tested how often the shortest standardized Euclidean distance between a trial’s data and the average of the training set predicted the location of the stimulus, separately for target and distractor location. In both data sets, we show that a single repetition of a distractor and target to the same location reduced decoding accuracies (Fig. 5a–b). Differences in distractor location decoding extended between 0.178 and 0.220 s (*p* = .002), while target location repetition effects diminished two clusters between 0.104 and 0.168 s (*p* = .004) and 0.188 and 0.236 s (*p =* .012), with the first cluster potentially corresponding to the P1 time period. A similar pattern of effects was observed in the EEG data (Fig. S6A–B).

Next, we investigated the effect of trial repetitions of stimulus features on neural representation. To do so, we first compared the standardized Euclidean distance between trials relative to the distractor orientation angle. As the similarity between two trials should scale with the similarity in angle, we were able to extract a tuning curve to the distractor angle over time, represented as heat maps (Fig. 5c). Clearly visible after 100 ms, we observe tuning to the distractor angle denoted by a higher similarity in red for similar angle trials compared to a lower similarity in dissimilar angles shown in blue. To quantify the tuning to orientation, we compared the tuning curve to a cosine function. This resulted in one value estimating the level of feature encoding over time (Fig. 5d). A cluster-based permutation test of all subjects’ tuning curves over all trials revealed significant encoding of stimulus features from 100 ms (0.098–0.378 s, *p* < .001), with peak tuning in MEG at around 200–300 ms. Finally, we focused on this peak tuning window, comparing mean cosine similarity for stimulus repetitions compared to nonrepetitions (Fig. 5e). In two independent comparisons of means, we found that distractor repetition resulted in diminished tuning to orientation, *t*(15) = 2.30, *p* = .036, while target repetition, as a control comparison, did not result in a significant reduction of distractor orientation coding, *t*(15) = 1.56, *p* = .139. However, both repetition conditions showed a similar pattern, with MEG being more attuned to repetition vs. nonrepetition effects and almost identical mean tuning values for each condition across the two analyses. EEG showed a similar pattern of effects although the reduced decoding of distractor orientation did not reach significance (Fig. S6D). Given that MEG and EEG are sensitive to different underlying neural sources, high decoding performance in MEG does not mean that we should always expect corresponding high decoding performance in EEG (Cichy & Pantazis, 2017). Therefore, this discrepancy between the two methods does not undermine findings in MEG.

## Discussion

Goal-directed behaviour is facilitated when our environment is consistent and we can anticipate patterns in our sensory processing. Here, we teased apart the contributions of stimulus repetition and higher order expectation of task relevant and task irrelevant information on behavioural performance and neural signals. We show subjects were faster and typically more accurate with increased expectations for both types of stimuli and that these effects were driven by expected repetitions in both cases. The behavioural similarity is not however mirrored in the neural measures. While early neural signals like the P1 were driven predominantly by low level repetitions, higher order expectations of targets and distractors were captured in decreased voltages and theta power in later frontocentral locations. However, we observed that a single repetition of a distractor is sufficient to reduce decodability of stimulus spatial location and is also accompanied by diminished representation of stimulus features.

Stimuli that are irrelevant to our goals can capture our attention and impair performance (Luck et al., 2021). Here, we show that increased expectation of these distracting stimuli can enhance performance in a comparable pattern to increased expectations derived from target repetitions. This pattern appeared robust and replicated across two studies and supports the growing literature on the behavioural value of being able to predict one’s environment, regardless of goal-relevance (Lamy et al., 2008; Reder et al., 2003, van Moorselaar et al., 2020; van Moorselaar & Slagter, 2019). Furthermore, we saw that both expected and unexpected repetitions improved behavioural performance for both targets and distractors and contributed unique variance to explain behaviour, thus replicating and extending findings from our previous work (Noonan et al., 2016). Collectively, these results suggest that repetition suppression and distractor expectation interact, with similar behavioural effects to those seen in the task-relevant stimulus domain (de Gardelle et al., 2013; Summerfield et al., 2011). In the context of the recently developed theories that spatial predictions of distractors and targets combine to form spatial priority maps (Wang & Theeuwes, 2018a, 2018b; Zhang et al., 2019), our results suggest that the initial stimulus repetition is particularly informative to this temporally evolving map. In our study, later repetitions appear to contribute less and less weight to the priority maps, although sensitivity for past repetitions may bridge discontinuities in expectations. Interestingly, our results suggest priority maps could be much more flexible than previous studies which showed that the longer term history, not the immediate past, most informs the priority map (Zhang et al., 2019) and that spatial distractor expectations are relatively robust to extinction (Valsecchi & Turatto, 2021). However, these inconsistencies likely reflect differences in the task-based volatility of stimulus spatial predictability, with the studies showing longer term influences holding the location of the frequent distractor constant for hundreds (sometimes thousands) of trials. By contrast, spatial predictability of a stimulus to a particular location in the present study repeatedly changed after approximately 30 trials. Potentially, therefore, subjects are learning both the spatial and temporal dynamics of the environment, which suggests that in volatile environments inhibitory mechanisms may adapt flexibly and rely on shorter term fluctuations in stimulus repetition to build saliency maps compared to tasks with less environmental volatility. Sensitivity to this higher order temporal structure is akin to dynamic learning rate mechanisms that track volatility of reward (Behrens et al., 2007). Our modeling results similarly suggest equivalent weighting of new spatial information contribute to the build-up of expectation for both target and distractor stimuli. Future research should examine the conditions and environmental factors that affect how stimulus expectations update spatial priority maps.

While our behavioural analyses broadly suggest similar patterns of processing for target and distractors, our assessment of neural EEG signals delineated the perceptual processing of the two stimuli. For example, the P1 demonstrated complementary but opposing processing of task-relevant and task-irrelevant information. Here, repetition of a target increases the P1 amplitude contralateral to target locations, whereas repetition of distractor produces the opposite effect, with decreased amplitude over electrodes contralateral to the distractor location. The P1 is thought to reflect the cost of attention (Luck et al., 1994) with decrements of this component occurring when attention is incorrectly focused outside of the target stimulus (Mangun & Hillyard, 1991; Van Voorhis & Hillyard, 1977), presumably, in our case, the distractor. The P1 suppression may represents the cost of stopping and shifting attention to the target location. As we found nothing to indicate the P1 amplitude was further modulated by the higher order expectations, our interpretation of the repetition effects falls in line with these views. Specifically, our results suggest that a repetition of a distractor is associated with a reduced neural attentional cost, while repetition of a target to the same location is associated with a neural attentional gain.

While the P1 has this bidirectional characteristic, suggesting complementary task-relevant and irrelevant neural mechanisms, the spatiotemporal and lateralized analyses suggest that other ERP components more clearly distinguish the two visual events. Only after a target repeats do we see that the P1 cluster of activity, localized contralaterally to the target in posterior sensors, advances anteriorly at the N1 time period, with a focus on lateralized occipitoparietal electrodes. Luck and colleagues (Luck et al., 1994; Vogel & Luck, 2000) have argued that the N1 indexes the benefit of attention and discrimination at attended locations, with increments in amplitude observed when attention is focused on a location where the target subsequently appears. The presence of target repetition effects over the N1 and not distractor repetition effects are in accord with this proposed role. By contrast, distractor repetition vs. nonrepetition only affects later timepoints, where we observe that the posterior visual signal ipsilateral to distractor locations spreads anteriorly into a large frontocentral cluster. This lateralized component likely corresponds to the N2pc, a component that reflects attentional selection (Eimer, 2014; Luck, 2012; Luck & Hillyard, 1994) and thus may reflect less attentional capture by the distractor. This pattern replicates our previous work, of which we further replicate the core findings with analogue analysis methods in the current data set (Noonan et al., 2016). The nature of our task prevents us from disentangling the N2pc from the Pd component, which has been argued to reflect attentional suppression. Not only has recent work shown that the magnitude of behavioral suppression reflects the magnitude of the PD component across subjects, distractor expectations of location and stimulus features are also capable of eliminating the PD component altogether (Gaspelin & Luck, 2018—although note this is not always the case; Feldmann-Wustefeld & Schubo, 2016).

In examining the differential effects of higher order distractor and target expectation in the present experiment, we distinguish between low-level repetition effects. We identified negative correlations between amplitude and increasing expectations of target and distractor throughout the trial. Specifically, increasing target expectation led to prolonged reductions in amplitude, beginning early in frontocentral electrodes and spreading posteriorly from there. By contrast, distractor expectation signals begin more posteriorly before rapidly localizing on frontal electrodes and then spreading posteriorly to broadly cover the scalp. This pattern in EEG amplitude is mirrored by frontocentral theta power, with theta negatively correlated with distractor and target expectation over the same time period. However, theta appears to be modulated both by repetition and expectation. Frontocentral theta has previously been linked with target expectation (Rungratsameetaweemana et al., 2018; Summerfield et al., 2011), with Summerfield and colleagues (2011) showing that expected repetition of face stimuli was associated with reduced theta relative to trials in which the face was expected to repeat but did not. Theta power did not distinguish between repetition and nonrepetition trials in a condition where faces were expected to alternate (equivalent to our 25% expectation condition). In the present study, we replicated Summerfield’s findings in the spatial domain with reduced theta to target repetition only if the target is expected. By contrast, we found that distractor repetition, regardless of expectation, was associated with reduced frontocentral theta power. With frontocentral theta power linked elsewhere to distractor inhibition in the context of conflict monitoring and resolution (Haciahmet et al., 2021; Zavala et al., 2013), we could infer here that conflict between target and distractor processing is reduced after both stimulus repetition and increased high-level expectation of a distractor.

Our results also suggest that stimulus evoked alpha power may have a role in modulating stimulus processing of expected and unexpected events and may distinguish between stimuli on the basis of task relevance. While preparatory alpha modulation and lateralization in anticipation of expected targets is well documented (Noonan et al., 2016; Worden et al., 2000), alpha lateralization is also triggered by the appearance of a target that cannot be anticipated, suggesting that alpha may also serve as an active mechanism of target processing (Bacigalupo & Luck, 2019). Interestingly, in line with current findings, stimulus evoked alpha lateralization also itself appears to depend on whether expectations of a target are violated or not (Rungratsameetaweemana et al., 2018). In this context, the present results may suggest that alpha is gating distractors that fulfill expectation through towards the next level of information processing via reduced alpha contralateral to these stimuli. By contrast, the absence of alpha lateralization after distractors that violate expectations potentially suggests that relative increased alpha contralateral to distractors reflects a stimulus evoked suppression. While this may be counterintuitive to the idea that expected distractors should be gated as quickly as possible to reduce the computational cost, it could be in line with the perceptual load theory (Lavie et al., 2004), with expected distractors potentially contributing less to perceptual load and thus being processed more.

Despite the collective evidence of reduced visual processing of distractor stimuli, thus far there remains the possibility that this reduced signal is accompanied by sharpened neural representations. In the target domain, if a presented stimulus feature matches one’s expectation then BOLD signal is reduced but classification accuracy increases, potentially reflecting sharpened neural tuning (Kok et al., 2012), with this increased sensitivity even evident during preparatory target feature processing (van Moorselaar et al., 2020; van Moorselaar & Slagter, 2019). Yet this pattern is not evident in distractor processing. Recent work has shown that, while distractor stimulus features can be decoded both in preparatory activity and stimulus evoked activity, they appear reduced when distractors are more predictable (van Moorselaar et al., 2020; van Moorselaar & Slagter, 2019). In line with this latter result, we also observed reduced classification accuracy for distractor features after a distractor repetition, suggesting that when a distractor stimulus repeats, there is broader tuning profile and thus a less defined neural representation of that distractor. By contrast, we saw no such reduction in distractor orientation decoding after a target stimulus repeated in the environment. Interestingly, though, while we could decode both distractor and target location, accuracy of decoding was reduced for both stimulus types after a single stimulus repetition to the same location of each respective class. This result could potentially be an index of response adaptation or a poorer neural representation of both stimuli. However, while the reduced target location decoding accuracy began early in visual processing, around the P1 time component, the distractor location decoding accuracy was diminished consistently at a later time window. One potential feature that may warrant future attention is the bimodal structure of the decoding curves. Notably, the nonrepetition decoding curves have a more pronounced structure of two peaks, one between 100 and 120 ms and another from 200 ms poststimulus. This possibly suggests that, after initial processing, stimulus information is refreshed after one alpha cycle (about 100 ms), and that this alpha refresh is more prominent when the information is more irrelevant—that is, on repeat trials, acting to locally suppress content. While past studies of our own work did not identify changes in alpha after distractor repetition, only target repetition (Noonan et al., 2016), more recent work has shown that alpha does track distractor location (Wöstmann et al., 2019), which may fit with this local mechanistic interpretation. Finally, we acknowledge the slight differences in decoding results between MEG and EEG. In addition to EEG being known as a noiser signal, with poorer spatial resolution than MEG, we note that these neuroimaging methods are sensitive to different underlying neural sources and so high decoding performance in one method should not lead to the expectation for corresponding high decoding performance in the other. While others have observed convergent and complementary decoding effects in the two methods, the same paper also noted neural differences contributed to decoding patterns (Cichy & Pantazis, 2017). This means that the discrepancy between the results in the present study should not undermine the positive effects we see in the MEG data, which, in the interests of transparency, should continue to motivate the adoption of multimodal analyses.

## Supplementary information

ESM 1 (DOCX 500 kb)
