# Supplementary material for: Differential modulation of visual responses by distractor or target expectations
Source: Atten Percept Psychophys. 2022 Dec 2;85(3):845–62. doi: 10.3758/s13414-022-02617-w (PMC10066164; doi:10.3758/s13414-022-02617-w)
Supplement: Supplementary file 1 — (DOCX 500 kb) [file 13414_2022_2617_MOESM1_ESM.docx]

Supplementary Material

EEG accuracy data and MEG RT and accuracy data

We replicated the behavioural analysis described in the Methods section for the RT and accuracy data collected from the MEG study. Replicating our findings from the EEG study Figure S1 suggests that RTs decrease with increasing expectation for targets and distractors and that this effect is driven by expected repeats for both task-relevant and irrelevant stimuli. Two independent repeated measures ANOVAs confirmed this pattern for the low (25%) and high (75%) Spatial Predictability conditions. RTs are reduced when targets repeat to the same location (F_1,15_ = 16.3, p = 0.001), although in this data set this does not vary as a function of Spatial Predictability (Repetition x Spatial Predictability F_2,30_ = 2.46, p = 0.138). The improvement in RT in repetition trials with increasing spatial predictability is confirmed by a one-way ANOVAs (F_2,30_ = 6.73, p = 0.004). The same statistical effects are evident for distractor processing (Repetition F_1,15_ = 20.0, p < 0.001, Repetition x Spatial Predictability F_2,30_ = 7.22, p = 0.017). And while the improvement in RTs in distractor repetition across spatial predictability conditions followed the same pattern, it was not significant (F_2,30_ = 1.97, p = 0.157). The pattern is also similar in the accuracy data although it fails to reach significance (2 x 2 ANOVA all ps >0.139, one way Target F_2,30_ = 1.31, p = 0.282, Distractor F_2,30_ = 2.66, p = 0.087).

**
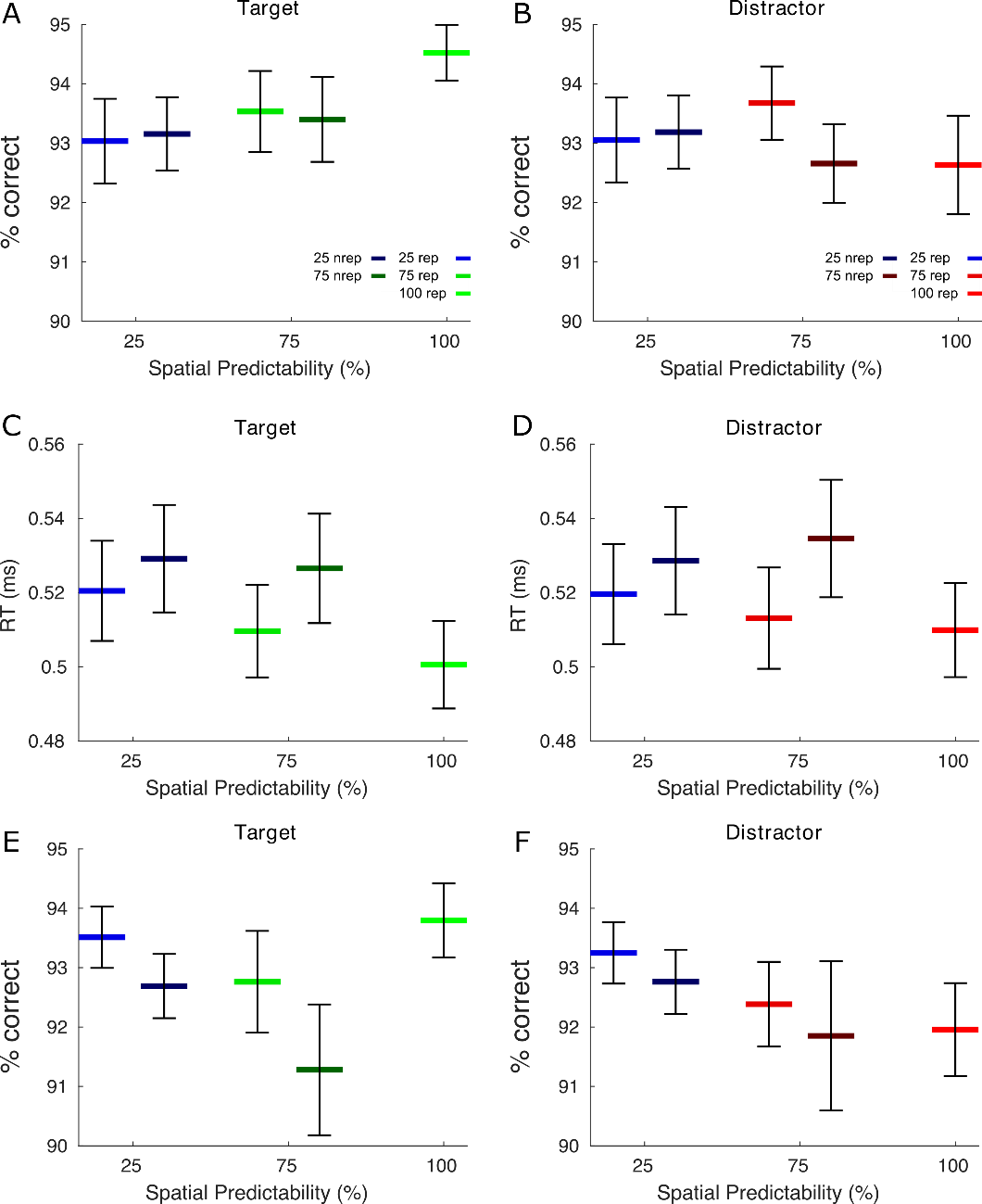
**

#### Figure S1. Impact of target and distractor expectation and repetition on accuracy for the EEG data and RT and accuracy in the MEG data. A. Mean and standard errors of percentage correct of target discrimination from the EEG study when the target has a 25%, 75% and 100% chance of appearing at a particular spatial location, for repetitions (rep, lighter colours) and non-repetition trials (nrep, darker colours) of the expected stimulus. The 25% condition is marked in blue to reflect the random nature of the stimulus repetition. B. Mean and standard errors of percentage correct when the distractor is the expected stimulus from the EEG study. Format is same as A. Treps and Dreps were independently pooled across the T25 and D25 conditions. While accuracy is mostly insensitive to differences between expected and unexpected repeats and non-repeats, there is a significant increase in accuracy with increasing target expectation. This pattern is not mirrored with increasing distractor expectation. See main text for full description of results. C & D. Mean and standard errors of RT from the MEG study in the same format as Figure 1C and D. E & F. Mean and standard errors of percentage correct from the MEG study in the same format as A and B here. Replicating the EEG study, the pattern suggests that RTs decrease with increasing expectation for targets and distractors and that this effect is driven by expected repeats for both task-relevant and irrelevant stimuli. Similarly, the accuracy data from the EEG follow the same behavioural trends seen in the MEG dataset.

Predictable versus unpredictable stimuli: Replication of past results

The data from the current experiment lends itself to be analysed in the same way as described in our previous study (Noonan, Adamian et al. 2016). To examine block-wise effects in Spatial Predictability of target and distractor, we pooled the data from T25 and D25 and compared this neutral condition (equivalent to the neutral cued trial in (Noonan, Adamian et al. 2016) to fully predictable T100 and D100 conditions. After artefact and trial rejection, one additional subject was excluded from this analysis because they had fewer than 20 trials in a condition. Previously we demonstrated reduced amplitude of the P1 contralateral to the distractor, and a loss of a significant N2pc when the distractor location is cued and repeated across a block of trials relative to neutral trials. We therefore hypothesised bilateral reduction in the P1 amplitude in distractor predictable conditions and therefore one-tailed t-tests were applied. Compared to the neutral trials, there is a significant reduction in the amplitude of the P1 contralateral to the distractor location in the fully predictable distractor blocks (t_28_ = 1.76, p = 0.045, one-tailed). Again, there is a trend towards a bilateral effect (ipsiD t_28_ = 1.71, p = 0.099, two-tailed). At later time points condition-wise differences suggest reduced bilateral amplitude when the target is fully predictable (contraT 0.288–0.328 secs, p = 0.047, ipsiT 0.256–0.320 secs, p = 0.009) and increased amplitude ipsilateral to the distractor when the distractor is fully predictable (ipsiD 0.268–0.300 secs, p = 0.033). Similarly, we isolated the N2pc, a lateralised components thought to reflect attentional selectivity to the target, by subtracting ipsilateral (to target) waveforms from contralateral ones. As previously shown when the distractor is fully predictable, it is less able to capture attention, as illustrated by a selective reduction in the amplitude of the N2pc (D100 vs TD25, 0.224–0.320 secs, p = 0.001). By contrast, amplitude contralateral to targets, when target stimuli are fully predictable, is significantly greater than ipsilateral channels (0.144–0.268 secs, p < 0.001). This suggests that these findings replicate even when spatial predictability develops implicitly.

**
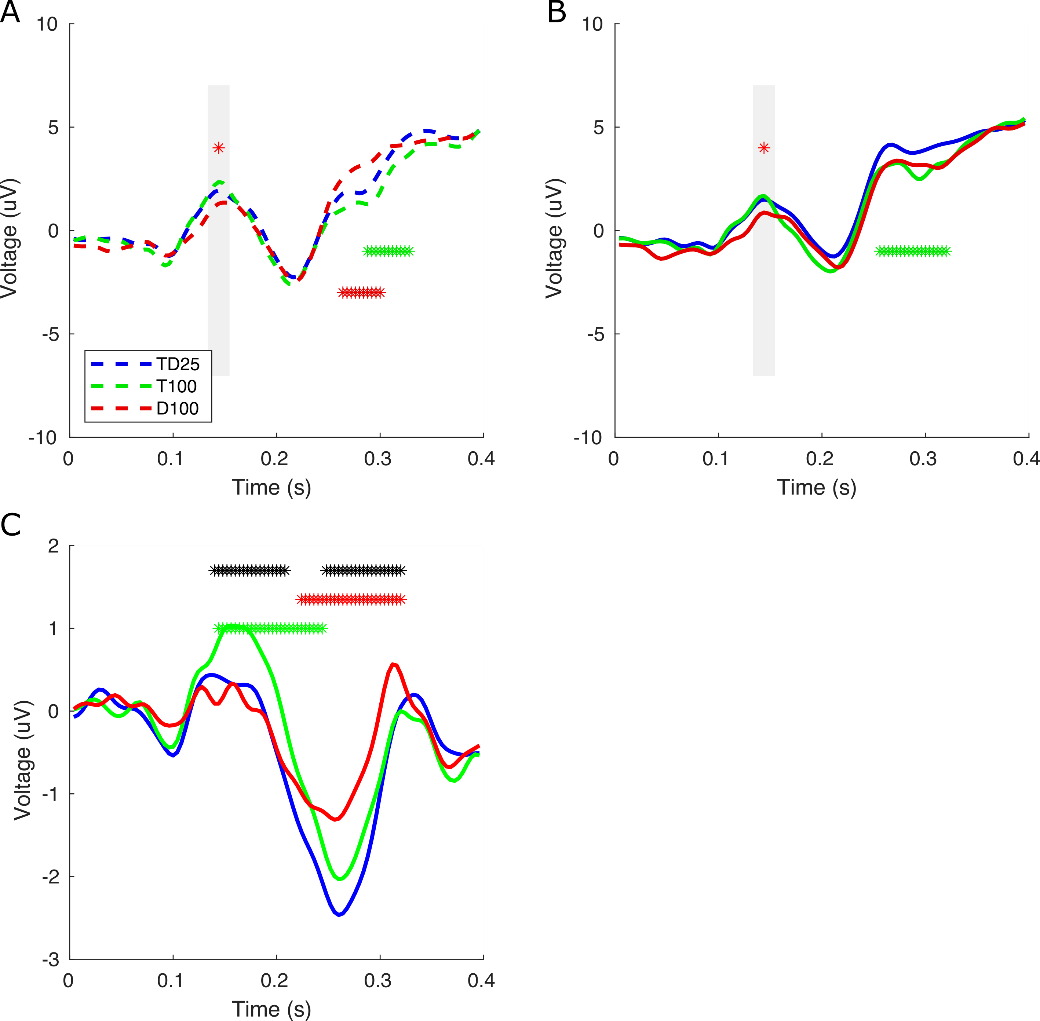
**

#### Figure S2. Distractor repetitions diminish P1 amplitude and N2pc: Replication of Noonan et al 2016. ERP waveforms for the fully expected target (T100, green), distractor (D100, red) and neutral (averaged across T25 and D25 = TD25, blue) conditions for sensors contralateral to target (contraT A, dashed lines) and ipsilateral to target (ipsiT, B, solid lines) when stimuli were presented in the upper visual field. Significance bar indicates cluster-corrected (p < 0.05) t-tests between expected and neutral trials (green represents neutral vs target; red represents neutral vs distractor). Gray panels represent a 20 ms window centered over the peak of the P1 component. Bilaterally, the P1 was reduced in amplitude when the distractor is fully predicable compared to the neutral condition. C. Lateralised components were isolated by subtracting ipsilateral waveforms from contralateral waveforms for each stimulus respectively. Fully expected distractors reduced the N2pc relative to neutral conditions (red asterisk: p < 0.001). Fully expected targets showed an enhanced earlier lateralised component in an extended significant cluster relative to neutral trials (green *: p = 0.001).

*Expectations for targets and distractors are updated independently*

To ultimately estimate higher order expectation for each stimulus on every trial we constructed and compared the fit of a number of reinforcement learning models to subject RT data. Specifically, learning rates (α) were fitted within the model individually for target and distractor to each subjects’ data using standard non-linear minimization procedures. Residual log-transformed RTs (calculated in the same way as for the RT regression analysis) were used to first estimate trial-wise expectations of each stimulus at each of the four locations. Residual RT was regressed against the spatial probability and minimized with the fmincon function on four stimulus events [1] target expectation in the target location (TT), [2] the target expectation at the distractor location (TD), [3] distractor expectation at the distractor location (DD) and [4] a distractor expectation at the target location (DT). Post-break, error and RT outlier trials were excluded from RT residual estimation but included in the initial model bootstrapping and final model fit. Four models were initially tested: [1] distractor learning rate was free to vary but target learning rate was fixed to 1 so expectation of subsequent distractor location was fully determined by the previous stimuli’s location, [2] target learning rate was free to vary but distractor learning rate was fixed to 1, [3] both target and distractor learning rates were fixed at 1, [4] both target and distractor learning rates were free to vary. A leave-one-out cross-validation procedure was used to compare model fits. The learning rates were minimised and fit to five of the six task blocks (128 trials per block) before the learning rates were applied to the untrained task block. Error fit for the test task block was estimated for each left out block for every subject and every model. All models started with equal stimulus expectation for each spatial location (0.25), learning rates of 0.2 for both stimuli, completing 1.0x10^8^ evaluations, and 1.0x10^12^ iterations. Mean error across task block fit was subjected to non-paramentric t-tests (Matlab signrank). The model struggled to fit one subject’s RT data so this subject with an implausibly low target learning rate and target expectations remaining around 0.25, which was therefore removed from the following model comparison analyses. Model comparisons suggested that model version four, where both learning rates could vary independently, best fit the data (Model 4 vs 1 z = 2.89, p = 0.0039, 4 vs 2 z = 2.87, p = 0.0042, 4 vs 3 = z = 4.49, p < 0.001).

The two optimized learning rates from the best fitting model version 4 were interrogated with paired t-tests and Pearson’s linear correlation coefficients. Interestingly, despite the differential relevance of the two expected stimuli, learning rates of target and distractors were not significantly different (t_28_ = 0.01, p = 0.992) and did not correlate within subjects (r = -0.13, p = 0.502). Further, while learning rates could be contributing valuable individual variance to the model fit, they could also simply reflect a noisy fit to each subject of the task mean. We explored this by creating another model version (model 5) that used model error estimates derived from a group average learning rate fit to all subjects’ data. We found that this group average model tended to significantly out-perform model 4 in fitting subjects RT data (z = -1.72, p = 0.086). We therefore used group average model 5 to estimate expectations and recover simulated residual RT estimates from the model ([Supplementary Figure 3](#_Figure_S1)). This also allowed us to re-introduce the subject with the poor model fit to the subsequent RT simulation and neural analyses.

Finally, as a last demonstration of model validity, we recovered residual RTs from model 5 and subjected this simulated data to the same behavioural analyses we performed on the real subject RT data. Using the beta values from the best-fit learning rates (glmval), model RT estimates were divided according to the same task condition labels in the three Spatial Predictability and two Repetition conditions and we conducted a 3 x 2 repeated measures ANOVA. Again, Trep trials in T25 and D25, and Drep trials in T25 and D25 were independently pooled. The results of this simulated analysis replicated the pattern of effects described above with decreasing RTs with increasing spatial predictability for both expected stimuli, with improvements particularly driven by more expected repetitions (Target; Repetition: F_1,29_ = 195, p < 0.001, Spatial Predictability: F_1,29_ = 187, p < 0.001, Interaction: F_2,58_ = 89.7, p < 0.001, one-way T25rep, T75rep, T100rep, F_2,58_ = 187.70, p < 0.001, Distractor; F_1,29_ = 87.2, p < 0.001, Spatial Predictability: F_1,29_ = 109, p < 0.001, Interaction: F_1,29_ = 56.8, p < 0.001, one-way D25rep, D75rep, D100rep, F_2,58_ = 90.94, p < 0.001).


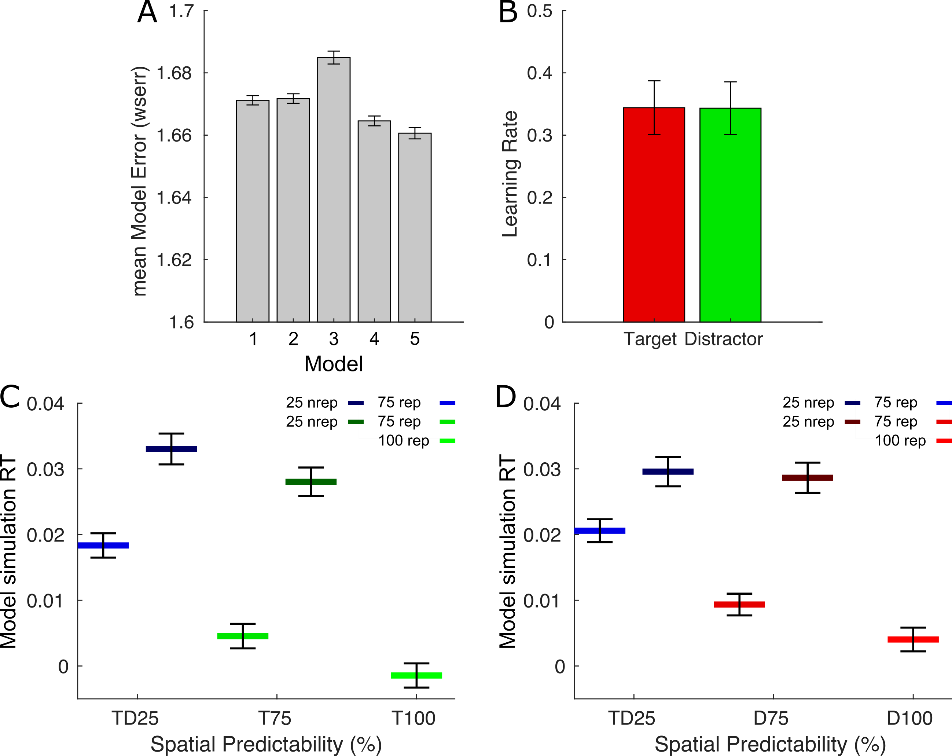


#### Figure S3. Model fits, learning parameters and simulated RT data. A. Mean model error (and standard error) for five reinforcement model fits. Models varied according to which learning rate parameter for target location expectation or distractor location expectation was free to vary and which were fixed to 1. In models 1 and 2, distractor and target learning rates were respectively allowed to vary, while the other stimulus learning rates were fixed. In model 3 both target and distractor learning rates were fixed at 1, while in model 4 both target and distractor learning rates were free to vary. Model 5 fit the data according to the group average of learning rates for target and distractors from model 4, suggesting that a model in which both learning rates can vary most closely fits the data. We show that the averaged group learning rates from model 5, when fit to the task blocks, are numerically a better fit than the individual subject and trend to a significantly better fit than model 4 (p = 0.086). [B] The learning rates for targets and distractors, as estimated from model 4, are not significantly different from each other. C. Model estimates of mean and standard errors of RTs for targets from the group average model 5. Simulated RT estimates are divided according to condition labels of the three levels of Spatial Predictability (25%, 75% and 100%) and Repetition (repeat vs non-repeat; rep lighter colours vs nrep darker colours) for the target (green) and distractor (red). The 25% condition in represented in blue to reflect the random nature of this condition. Treps and Dreps were independently pooled across the T25 and D25 conditions. D. Model estimates of mean and standard errors of RTs for distractors from the group average model 5, in the same format as C. Simulated model RT reduction with increased expectation was driven by expected stimulus repeats for both targets and distractors.

Target and distractor expectation during the P1 time window


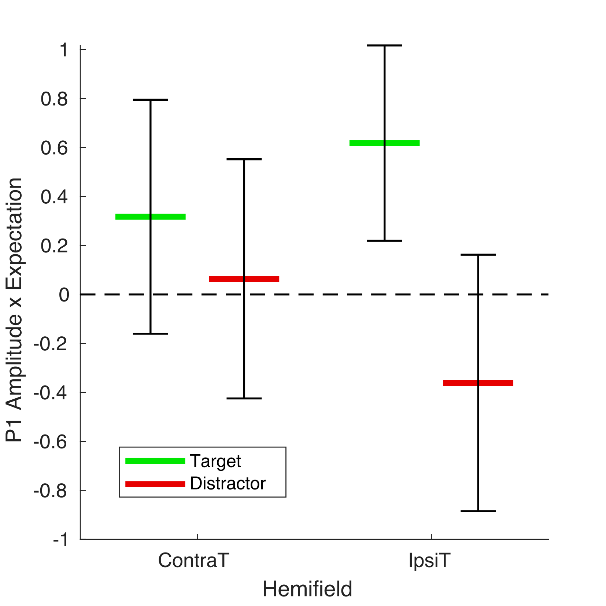


#### Figure S4. Amplitude of the P1 does not correlate with target or distractor expectation. Mean beta weights and standard error for a regression between extracted ERP amplitude at the P1 time window and trial-wise target (green) and distractor (red) expectation (with the variance associated with stimulus repetition removed), averaged over sensors contralateral (contraT) and ipsilateral (ispiT) to the target. While the direction of expectation effects are in line with the repetition effects, with increasing target expectation associated with increasing P1 amplitude and increasing distractor expectation associated with decreasing P1 amplitude, the effects are not significantly different from zero. This suggests that the effects at this early time point are predominantly driven by the expectation of a stimulus repetition and not necessarily linked to higher-order expectations. See main text for full results description.

Trial-wise stimulus repetition effects in frontocentral theta

Given the link between frontocentral theta power and target expectation (Summerfield, Wyart et al. 2011), we tested whether frontocentral theta distinguished between stimulus relevance (targets vs distractors). Log-transformed theta power (from C2, C1, C4, C3, CP4, CP3, CPz, Cz, FC3, FC1, FCz, FC4, FC2 channels) was compared across repetition and non-repetition trials in the high and low Spatial Predictability blocks (75rep vs 75nrep and 25rep vs 25nrep). For this analysis stimuli did not have to be lateralised. Statistical differences were estimated with cluster corrected t-tests using permutation-based statistics. We identified significant reductions in theta power for repeated expected targets (T75) between 0.224–0.376 secs (p = 0.021) relative to non-repeat targets, but there were no significant difference between theta power for unexpected target repeats (TD25, p = 0.124). By contrast, theta power was significantly reduced for both expected (D75, 0.216–0.392 p = 0.031) and unexpected (TD25, 0.104–0.312 secs p = 0.025) distractor repeats compared to trials in which the distractor did not repeat.


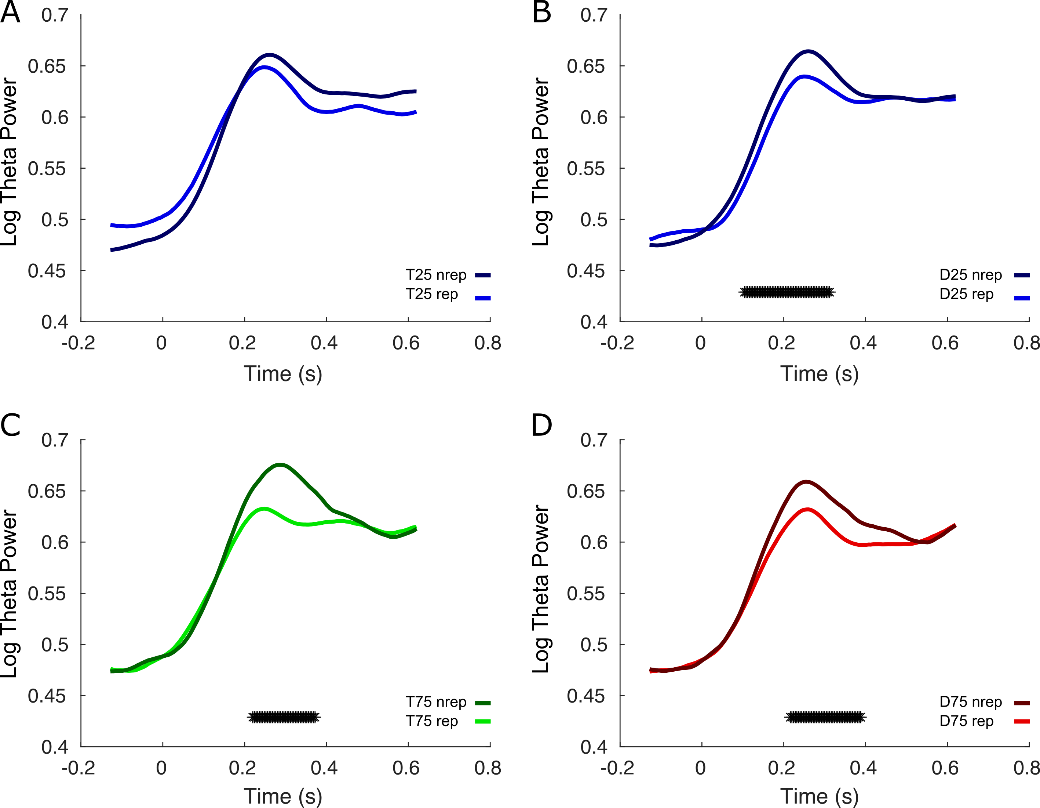


#### Figure S5. Stimulus repetition effects in frontocentral theta power distinguishes targets but not distractors. Mean log theta power pooled across T25 and D25 (TD25) conditions for target (A) and distractor (B) unexpected repeats (light blue) and respective non-repeats (dark colours). Mean log theta is also shown for expected target repeats (T75, C) and expected distractor repeats (D75, D) in light green and red respectively and their respective non-repeats in dark respective colours. Asterisks indicate significant differences between stimulus repetition and non-repetition trials using non-parametric permutation-based correction (p < 0.05). Significant cluster corrected reductions in theta power for repeated expected targets (T75) was found between 0.224–0.376 secs (p = 0.021) post-stimulus relative to non-repeat targets, but there were no significant differences between theta power for unexpected target repeats (TD25, p = 0.124). By contrast, theta power was significantly reduced for both expected (D75, 0.216–0.392 s, p = 0.031) and unexpected (TD25, 0.104–0.312 secs, p = 0.025) distractor repeats compared to trials in which the distractor did not repeat.

Decoding distractor location and orientation features in EEG

In parallel to the MEG analysis, we examined the EEG dataset using the same analysis decoding procedure. We found differences in distractor location decoding extended for a longer period of time than for target decoding, with target repetition effects only relevant between 0.092–0.016 secs (p = 0.001), potentially corresponding to the earlier P1 ERP components, while distractor repetition effects diminished in two clusters between 0.084–0.164 secs (p < 0.001) and 0.172–0.248 secs (p < 0.001) (Figure S6A,B). We also investigated the effect of trial repetitions of stimulus features on neural representation in the EEG data. Again, we compared the standardized Euclidean distance between trials relative to the distractor orientation angle. We extracted the tuning curve to the distractor angle over time (Figure S6C) with a clear tuning to distractor angle visible after 100 ms (higher similarity for similar angel trials in red). This was quantified by comparing the tuning curve to a cosine function that reflected the level of feature encoding over time (Figure S6D). Cluster-based permutation test of all subjects’ tuning curves over all trials revealed significant encoding of stimulus features after 100 ms, with peak tuning in EEG at between 0.088–0.400 secs (p = 0.002). Again, we focused on this peak tuning window and compared mean cosine similarity for stimulus repetitions compared to non-repetitions (Figure S5E) and report a non-significant trend towards diminished tuning to orientation after distractor repetition (t_29_ = 1.51, p = 0.141), mirroring the MEG data. Target repetition did not result in a significant reduction of distractor orientation (t_29_ = 0.55, p = 0.589).


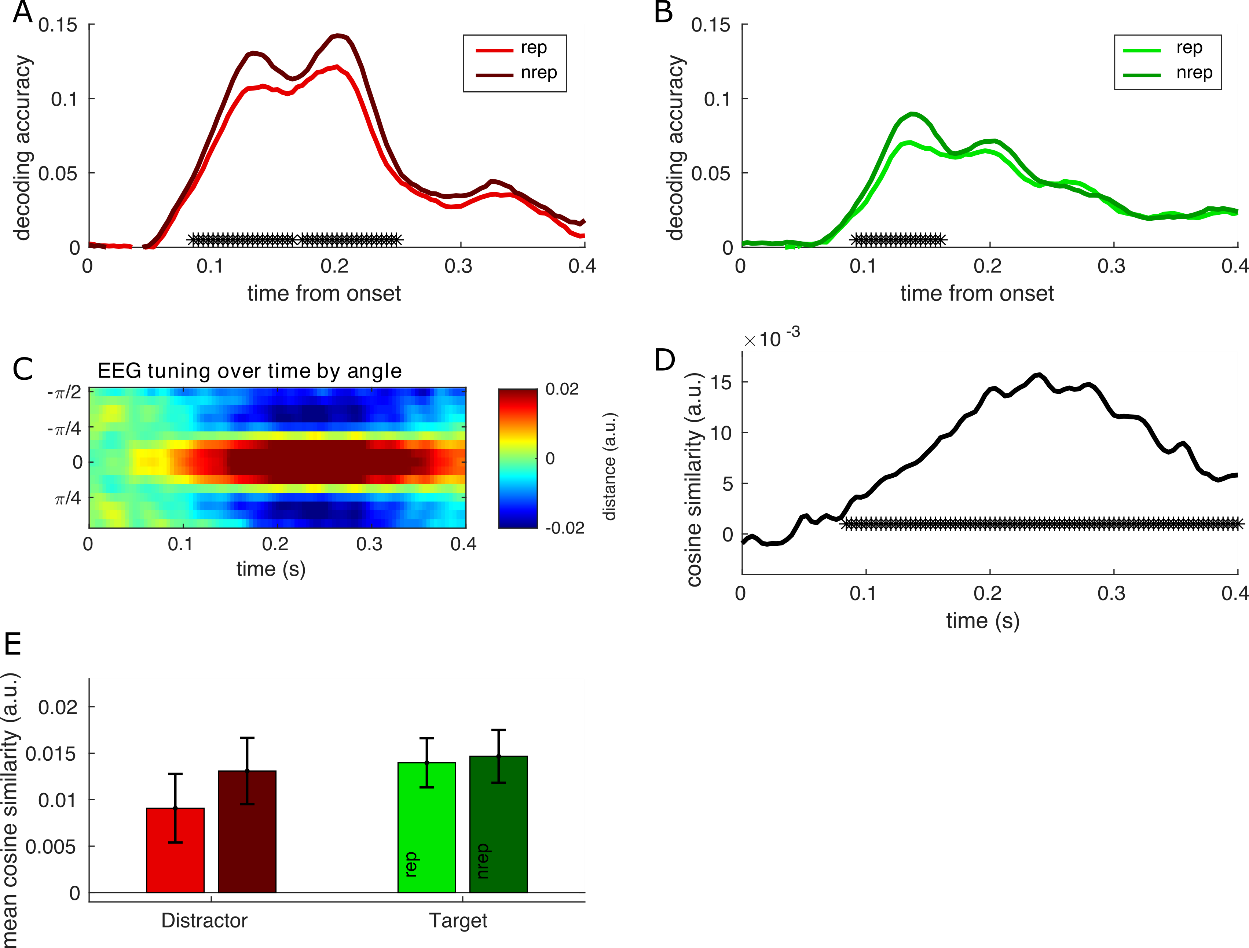


#### Figure S6. Representation of distractor location and stimulus features in EEG. Accuracy of decoding distractor (A) and target (B) spatial location is reduced after a single trial repetition (lighter coloured line) relative to a non-repetition trial (darker coloured line). Significant differences in distractor location decoding extend in two clusters between 0.084–0.164 secs and 0.172–0.248 secs while target repetition effects are significantly different between 0.092–0.016 secs (denoted by asterisks). C. Heat maps show mean standardized Euclidean distance between trials relative to the distractor orientation angle and represent a tuning curve to the distractor angle over time. Hot/red areas reflect higher similarity for similar angles while cold/blue areas represent lower similarity in dissimilar angles. Higher tuning to distractor angle is visible after 100 ms. D. Comparison of the tuning curve to a cosine function averaged across participants and representing feature encoding across time. Asterisks denote significant cluster-based permutation tests of subjects’ tuning curves and show significant stimulus features encoding with a peak between 0.088–0.400 secs. E. Mean and standard error of the cosine similarity extracted from the peak tuning window for stimulus repetitions and non-repetitions. The pattern of EEG mirrors the MEG analysis with a non-significant trend towards distractor repetition diminishing orientation tuning while target repetition does not show such an impact on distractor orientation tuning.
